# A conceptual model of factors potentially influencing prescribing decisions for chronic conditions: an overview of systematic reviews

**DOI:** 10.1186/s12916-025-04194-9

**Published:** 2025-07-01

**Authors:** Matthew Gittus, Alicia O’Cathain, Katherine Miller, Anja Oklopcic, Albert C. Ong, James Fotheringham

**Affiliations:** 1 University of Sheffield, Sheffield, UK; 2 Sheffield Kidney Institute, Northern General Hospital, Sheffield, UK

**Keywords:** Long-term medications, Chronic conditions, Chronic diseases, Prescribing, Decision-making

## Abstract

**Background:**

Nearly half of all adults are affected by chronic conditions with long-term medications often being the primary intervention. Although models like that of Murshid and Mohaidin contribute to our understanding of prescribing behaviours, they are not specific to chronic conditions and may not reflect the full range of influencing factors relevant to long-term care. Better understanding the factors that may influence healthcare professionals’ decision-making could help inform policy and guidelines as well as identify targets for future research and interventions.

**Methods:**

An overview of systematic reviews was undertaken, following the 2020 PRISMA guidelines. PubMed, Embase, Web of Science, Cochrane Library and Google Scholar were searched from 01/01/2013 to 7/11/2023. Quality assessment was undertaken using the AMSTAR 2 tool. Screening, data extraction and synthesis were conducted. Confidence in findings was assessed using the GRADE-CERQual tool. An existing generic conceptual model of prescribing was adjusted to specifically reflect chronic conditions.

**Results:**

Twenty-six reviews published between 2013 and 2023 were included, synthesising 689 primary studies. Patient factors that may influence prescribers’ decisions included age, ethnicity, education and level of rurality of residence. Prescribers describe assessing individual patient characteristics when weighing the risks and benefits, with a tendency to prioritise risks—especially for patients with multiple comorbidities or complex needs. Prescribers’ approach to risk may be influenced by their clinical experience, care setting and assessment tools. High workload and competing priorities may lead to clinical inertia in terms of delaying or preventing medication initiation. Shared decision-making may not always be shared equally between patients and prescribers. Beyond direct medication costs, prescribers may also consider broader healthcare costs, such as the need for monitoring and use of support staff for monitoring. External factors such as guidelines may be helpful in navigating risks, with their effectiveness potentially enhanced when they offer specific recommendations tailored to prescribers’ population characteristics.

**Conclusions:**

Prescribers may need to navigate multiple challenges when making prescribing decisions for people with chronic conditions. This overview of systematic reviews suggests possible interrelated factor categories influencing prescribing decisions. The conceptual model may be used as a framework for future research and development of interventions.

**Supplementary Information:**

The online version contains supplementary material available at 10.1186/s12916-025-04194-9.

## Background

Chronic conditions represent a significant and growing health issue, affecting nearly half of all adults [1, 2]. The World Health Organization defines them as “long duration, non-communicable” diseases caused by “a combination of genetic, physiological, environmental and behavioural factors” [3]. Conditions must persist for at least 6 months to be labelled chronic [4]. They are a leading cause of mortality, accounting for 74% of deaths and contribute to 85% of healthcare expenditure worldwide [3, 5]. The prescription of long-term medications is often the primary intervention to control disease progression, reduce complications and improve patient outcomes [6–8]. However, prescribing decision-making in chronic conditions is complex [9, 10] and may differ from that in acute conditions.

**Fig. 1:**
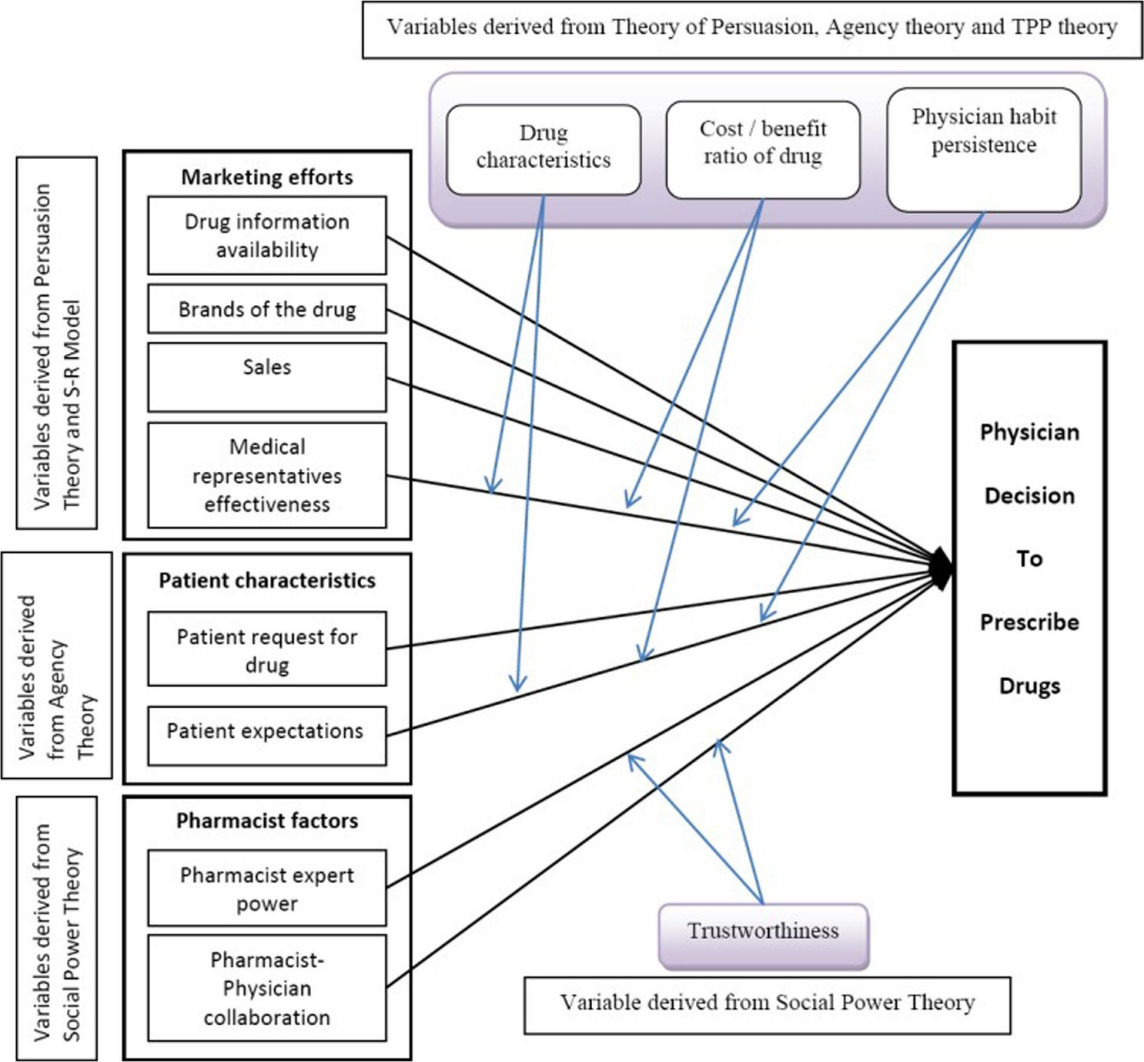
Conceptual model of physician decision-making process—image reproduced from Models and theories of prescribing decisions: a review and a suggested new model by Murshid M A and Mohaidin Z, published in *Pharmacy Practice (Granada)*, under the CC BY-NC-ND 3.0 licence

Prescribing decision models, such as the conceptual model by Murshid and Mohaidin (Fig. 1) [11–14], suggest several factor categories that may influence prescriber behaviour, including patient characteristics, pharmacist factors, marketing efforts and contextual factors, as well as trust between physicians and pharmacists. However, these models do not distinguish between prescribing in chronic and acute conditions, despite the different objectives of these prescribing contexts [15]. Acute prescribing may be more typically urgent and symptom-driven, focussing on immediate relief or resolution of illness [16]. Whereas, chronic prescribing often aims to slow disease progression, may require a more proactive approach and involves long-term monitoring [17]. Furthermore, existing models may not fully account for the influence of prescriber characteristics nor incorporate some additional factors identified in the wider literature such as clinical guidelines [18], disease severity or risk assessment scores [19] and clinical decision support systems [20]. Thus, there is a need to reassess existing models, with a specific focus on chronic conditions.

The existing literature on prescribing in chronic conditions includes systematic reviews describing factors that may influence prescribing decisions for specific medication classes or single disease types. Due to the breadth and complexity of these factors, decision-makers may struggle to navigate and synthesise the existing evidence. To address this, an overview of systematic reviews was conducted to provide a comprehensive overview of the factors that may influence prescribing practices. By collating, synthesising and summarising the available evidence, this overview aims to clarify key factors that may shape prescribing decisions for long-term medications in chronic conditions and provide a chronic condition-specific model. This model may help better inform policy development and identify potential targets for future research and interventions.

The objectives of this overview were to address the following research questions:

- What are the factors that may influence the prescribing of long-term medications for chronic physical health conditions?
- What changes, if any, are needed to an existing prescribing model by Murshid and Mohaidin to better reflect the factors that may influence prescribing for chronic conditions?

## Methods

### Study design and protocol

We conducted an overview of systematic reviews following guidance from Cochrane [21], Hunt et al. [22], Gates et al. [23] and the enhancing transparency in reporting the synthesis of qualitative research (ENTREQ) checklist [24]. The overview was reported according to the 2020 preferred reporting items for systematic review and meta-analysis (PRISMA) statement [25]. The protocol was registered on PROSPERO (CRD42023467276).

### Scope

This overview of systematic reviews synthesised systematic reviews of qualitative, quantitative and mixed methods studies of factors that may influence prescribing in chronic physical health conditions.

### Eligibility criteria

The eligibility criteria are included in Table 1 based on the SPIDER (Sample, Phenomenon of Interest, Design, Evaluation, Research type) framework [26].

**Table 1:** Eligibility criteria reported according to the SPIDER framework

**Sample**	Adults with non-communicable chronic physical health conditions. Children were excluded due to developmental and pharmacokinetic variations and a lower evidence base [27–29]. Chronic mental health conditions were not included as non-medication approaches are typically recommended as first-line treatment in these conditions [30, 31]. Communicable diseases (e.g. HIV, tuberculosis) were excluded as they are not classified as chronic, and treatment aims to prevent progression or transmission with less room for individualised prescribing [32]
Phenomenon of interest	Only factors influencing medication initiation were considered. Other stages of the prescribing process, such as titration and discontinuation, were not included as they are less commonly explored in the literature
Design	Published peer-reviewed systematic reviews. Other types of review including rapid reviews and scoping reviews were not included. Protocols and conference abstracts were not included
Evaluation	Eligible systematic reviews included those with results on at least one of the following outcomes relevant to prescribing decision-making: (i) Factors influencing initiation of long-term medications (ii) Experiences of healthcare professionals making prescribing decisions (iii) Barriers and facilitators to prescribing long-term medications
Research type	As described in the scope, systematic reviews of qualitative, quantitative and mixed methods studies published in any language between 01/01/2013 and 07/11/2023 were included

### Search strategy

A comprehensive search strategy, including automated and manual search techniques, was developed using the SPIDER framework [26]. The following databases were searched in line with Bramer et al.: PubMed MEDLINE (including electronic publications ahead of print), EMBASE, Web of Science (Core Collection), Cochrane Library and Google Scholar (the first 200 relevant references) [33]. Reference checking and citation searching were implemented manually to complement the initial search strategy [34]. Preliminary searches using the terms “chronic condition*” or “disease*” produced an overwhelming number of irrelevant results. To refine the search, a list of chronic diseases generated by 83 stakeholders from an existing systematic review on self-management support for people with chronic conditions was incorporated into the search strategy (Additional file 1: Table 1) [35]. Protocols and conference abstracts were not included at the full-text stage but a manual search was undertaken to identify any subsequent published reviews. Forwards and backwards citation searching for other reviews was conducted on the included reviews. Searches were conducted from the 1 st of January 2013 to the 7th of November 2023 to ensure that the findings were relevant to current clinical practices and policies. There was no time limit for primary studies in the included reviews. There were no language restrictions. The full search strategy is in Additional file 2: Tables 2–6.

### Selection of systematic reviews

The search results were imported into HubMeta; duplicates were eliminated through the automated deduplication tool and confirmed manually by one reviewer (MG). Titles and abstracts were independently reviewed by any two of three reviewers (MG, AO and KM) to evaluate eligibility against the pre-specified criteria. An initial calibration exercise was undertaken between reviewers using a random sample comprising 1% of the search results. Full texts of all reviews identified as potentially relevant by both reviewers were retrieved and independently assessed for eligibility by each of the two main reviewers (MG and KM). Any discrepancies were resolved through discussion. If discrepancies persisted, consultation with another member of the research team was sought (JF).

### Data extraction and assessment of methodological limitations

Data was extracted from each systematic review: study settings, data collection methods, methodology and study design. For the systematic reviews with qualitative findings, factors from the Murshid and Mohaidin conceptual model [12] were listed on the data extraction form to ensure systematic extraction of qualitative findings. If new factors were identified, they were added to the list of factors [12]. Factors identified in the systematic reviews with quantitative findings were then extracted. One reviewer (MG) extracted relevant data, which was subsequently verified by a second reviewer (KM) for accuracy.

### Quality assessment

To assess the quality of the included systematic reviews, the AMSTAR 2 (A Measurement Tool to Assess Systematic Reviews 2) tool was employed [36]. This tool evaluates the methodological quality of systematic reviews based on 16 domains [36]. Systematic reviews were scored independently by two reviewers (MG and KM) with any discrepancies addressed through discussion. If discrepancies persisted, consultation with another member of the research team (JF) was sought. Although AMSTAR 2 is not intended to generate an overall score, systematic reviews with significant weaknesses across critical domains may raise concerns regarding confidence in their findings [37]. Studies that lacked methodological rigour—if they scored more than 5 critical domain weaknesses—were excluded.

### Overlap analysis

Overlap analysis in overviews of reviews examines the extent to which primary studies appear across multiple systematic reviews. The corrected covered area metric, ranging from 0 (no overlap) to 1 (complete overlap), was used [38].

### Synthesis

Synthesis was undertaken by one reviewer (MG) in discussion with co-authors (JF and AOC). Qualitative research in this context explores perceptions of stakeholders concerning influences on their prescribing. Factors perceived as influencing the prescribing of long-term medications for chronic conditions identified in the qualitative research in the systematic reviews were synthesised narratively. Themes were identified using the “framework” approach by reading the systematic reviews, identifying a thematic framework and coding each review to that framework [39, 40]. Murshid and Mohaidin’s conceptual model was used as a thematic framework and added to as coding progressed [12].

Quantitative research in this context identifies factors that have been tested by researchers for association with prescribing behaviours. The factors tested are usually limited to those that are available to researchers or easily measured. The study designs used can identify association but not causation. Factors identified in the quantitative research in the systematic reviews were synthesised using a vote counting approach based on direction of effect [41].

#### Confidence in findings

The GRADE-CERQual tool was used to assess confidence in findings. One reviewer (MG) conducted GRADE-CERQual assessments based on four components: methodological limitations, coherence, adequacy of data and relevance. Each component was assessed by the level of concern (no or very minor, minor, moderate or serious). A judgement was made about the overall confidence in review findings (high, moderate, low or very low). Findings were initially assigned high confidence, with downgrades applied if notable concerns were identified within any of the four GRADE-CERQual components [42].

#### Constructing the new conceptual model

Factors related to the themes identified from qualitative research in the systematic reviews were incorporated into the conceptual model by Murshid and Mohaidin and a diagram produced.

## Results

**Fig. 2:**
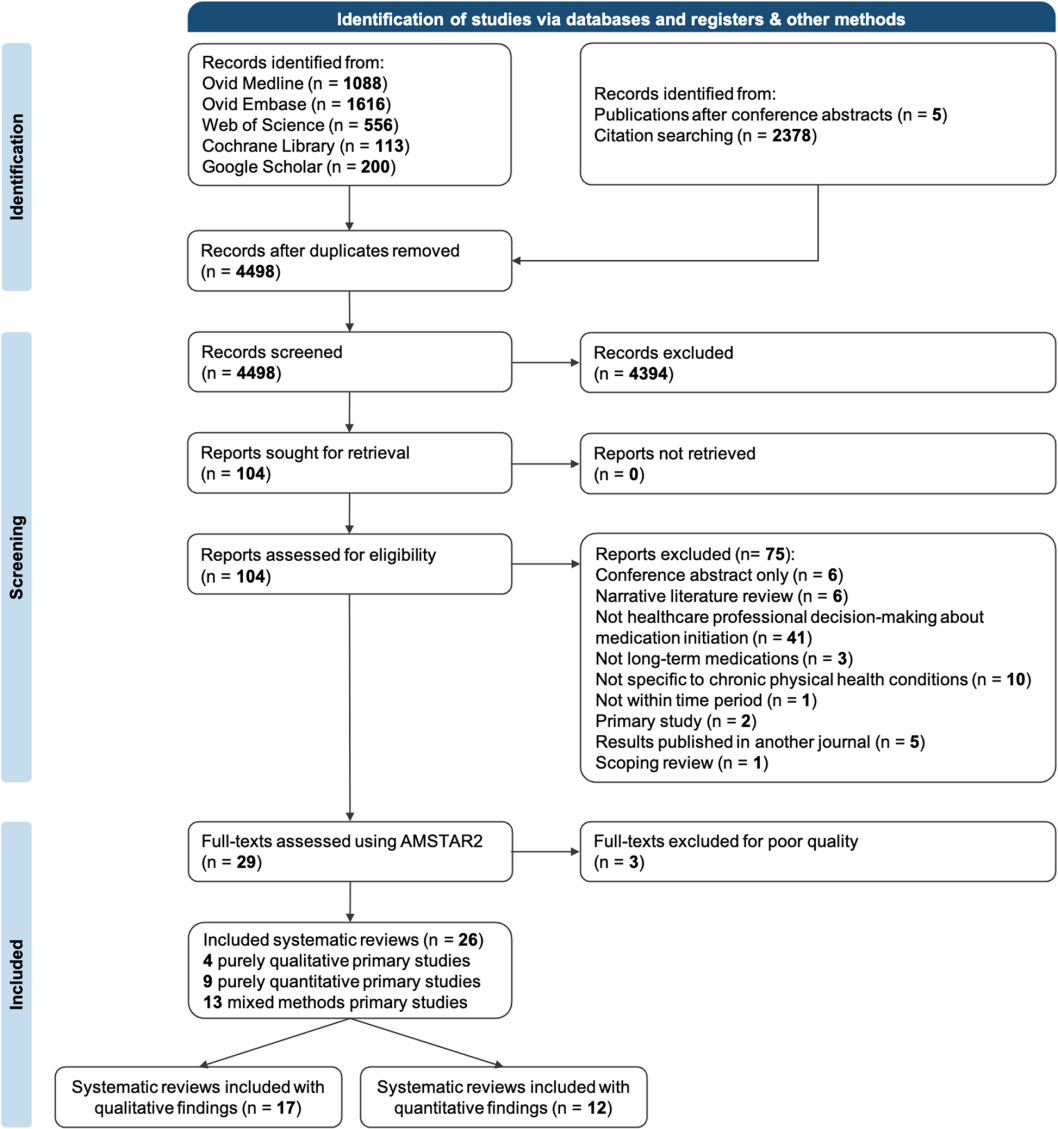
PRISMA flowchart

A total of 4498 articles were identified from database searches. One hundred four full-text articles were assessed for eligibility and 26 systematic reviews were included (Fig. 2). For a detailed version of the PRISMA diagram, see Additional file 3: Fig. 1.

The 26 included systematic reviews had lead authors from 10 countries (see Table 2). Most reviews were conducted by authors in high-income countries and were published towards the latter part of the 2013 to 2023 period. Four reviews included only qualitative primary research studies, nine reviews included only quantitative primary research studies and 13 included both types and mixed methods studies. Cumulatively they included 689 primary research studies, ranging from 3 to 69 primary studies per review. Although the inclusion criteria and search strategy encompassed any prescribers, all of the included studies focussed on factors that may influence physicians’ prescribing practices. For further details of the included systematic reviews, see Additional file 4: Table 7.

**Table 2:** Characteristics of included systematic studies

Authors (year)	Title	Country of first author	Condition	Medication	Methodology	Studies
**Abdelkader et al. (2023) ** [43]	Prescribing patterns of antihypertensive medications: a systematic review of literature between 2010 and 2020	Qatar	Hypertension	Antihypertensives	Mixed methods	40
**Arshad et al. (2021) ** [44]	Prescribing patterns of antihypertensive medications in low- and middle-income countries: a systematic review	Pakistan	Hypertension	Antihypertensives	Quantitative	26
**Bin Rsheed and Chenoweth (2017) ** [45]	Barriers that practitioners face when initiating insulin therapy in general practice settings and how they can be overcome	Saudi Arabia	Diabetes mellitus	Insulin	Mixed methods	19
**Byrne et al. (2022) ** [46]	Individual, healthcare professional and system-level barriers and facilitators to initiation and adherence to injectable therapies for type 2 diabetes: a systematic review and meta-ethnography	UK	Diabetes mellitus	Insulin and injectable hypoglycaemic agents	Qualitative	42
**Chin et al. (2016) ** [47]	The treatment gap in patients with chronic systolic heart failure: a systematic review of evidence-based prescribing in practice	Australia	Cardiovascular disease	Heart failure medications	Quantitative	23
**Dhungana et al. (2021) ** [48]	Barriers, enablers and strategies for the treatment and control of hypertension in Nepal: a systematic review	Australia	Hypertension	Antihypertensives	Mixed methods	15
**Generalova et al. (2018) ** [49]	A systematic review of clinicians’ views and experiences of direct‐acting oral anticoagulants in the management of nonvalvular atrial fibrillation	UK	Atrial fibrillation	Anticoagulants	Mixed methods	10
**Iudici et al. (2014) ** [50]	Prevalence and factors associated with glucocorticoids (GC) use in systemic sclerosis (SSc): a systematic review and meta-analysis of cohort studies and registries	Italy	Systemic sclerosis	Glucocorticoids	Quantitative	23
**Ju et al. (2018) ** [51]	General practitioners’ perspectives on the prevention of cardiovascular disease: systematic review and thematic synthesis of qualitative studies	Australia	Cardiovascular disease	Preventative cardiovascular agents	Mixed methods	34
**Khatib et al. (2022) ** [52]	Patient and healthcare provider barriers to hypertension awareness, treatment and follow up: a systematic review and meta-analysis of qualitative and quantitative studies	Canada	Hypertension	Antihypertensives	Mixed methods	69
**Lalor et al. (2022) ** [53]	Factors influencing clinician prescribing of disease-modifying anti-rheumatic drugs for inflammatory arthritis: a systematic review and thematic synthesis of qualitative studies	Australia	Inflammatory arthritis	Disease modifying anti-rheumatics	Qualitative	15
**Mahmoud et al. (2023) ** [54]	Meta-analysis of factors associated with antidiabetic drug prescribing for type 2 diabetes mellitus	UK	Diabetes mellitus	Oral hypoglycaemics	Quantitative	40
**Maimaris et al. (2013) ** [55]	The influence of health systems on hypertension awareness, treatment, and control: a systematic literature review	UK	Hypertension	Antihypertensives	Mixed methods	53
**Mas Dalmau et al. (2017) ** [56]	Patients’ and physicians’ perceptions and attitudes about oral anticoagulation and atrial fibrillation: a qualitative systematic review	Spain	Atrial fibrillation	Anticoagulants	Mixed methods	9
**Milman et al. (2018) ** [57]	Clinical inertia in the pharmacological management of hypertension: a systematic review and meta-analysis	Canada	Hypertension	Antihypertensives	Quantitative	8
**Ng et al. (2015) ** [58]	Barriers and facilitators to starting insulin in patients with type 2 diabetes: a systematic review	Malaysia	Diabetes mellitus	Insulin	Mixed methods	25
**Oqab et al. (2018) ** [59]	What is the impact of frailty on prescription of anticoagulation in elderly patients with atrial fibrillation? A systematic review and meta-analysis	Canada	Atrial fibrillation	Anticoagulants	Quantitative	3
**Orayj and Lane (2019) ** [60]	Patterns and determinants of prescribing for Parkinson’s disease: a systematic literature review	UK	Parkinson’s disease	Dopaminergic agents	Mixed methods	44
**Osasu et al. (2021) ** [61]	Patients’ and clinicians’ perceptions of oral anticoagulants in atrial fibrillation: a systematic narrative review and meta-analysis	UK	Atrial fibrillation	Anticoagulants	Mixed methods	34
**Pokorny et al. (2022) ** [62]	Interactions with the pharmaceutical industry and the practice, knowledge and beliefs of medical oncologists and clinical haematologists: a systematic review	Australia	Cancer	Chemotherapy/haematological agents	Mixed methods	31
**Presta et al. (2022) ** [63]	Impact of frailty models on the prescription of oral anticoagulants and on the incidence of stroke, bleeding, and mortality in older patients with atrial fibrillation: a systematic review	Italy	Atrial fibrillation	Anticoagulants	Quantitative	23
**Pritchett et al. (2020) ** [64]	Clinicians’ views and experiences of prescribing oral anticoagulants for stroke prevention in atrial fibrillation: a qualitative meta-synthesis	UK	Atrial fibrillation	Anticoagulants	Mixed methods	13
**Proietti et al. (2022) ** [65]	Frailty prevalence and impact on outcomes in patients with atrial fibrillation: a systematic review and meta-analysis of 1,187,000 patients	Italy	Atrial fibrillation	Anticoagulants	Quantitative	33
**Qadi et al. (2020) ** [66]	Patients’ and health professionals’ attitudes and perceptions towards the initiation of preventive drugs for primary prevention of cardiovascular disease: a systematic review of qualitative studies	UK	Cardiovascular disease	Preventative cardiovascular agents	Qualitative	5
**Rushforth et al. (2014) ** [67]	Barriers to effective management of type 2 diabetes in primary care: qualitative systematic review	UK	Diabetes mellitus	Injectable and oral hypoglycaemic agents	Qualitative	32
**Wilkinson et al. (2019) ** [68]	Management of atrial fibrillation for older people with frailty: a systematic review and meta-analysis	UK	Atrial fibrillation	Anticoagulants	Quantitative	20

The “corrected covered area” was 0.03, indicating little overlap among the primary studies in the included systematic reviews. 65.4% (17/26) of the included reviews focussed on cardiovascular conditions (cardiovascular disease, atrial fibrillation and hypertension). Inter-rater reliability at the title/abstract stage of screening was 0.65 (Cohen’s kappa) and at full-text stage was 0.89 (Cohen’s kappa).

For the list of excluded systematic reviews at the full-text stage and the reasoning behind their exclusion, see Additional file 5: Table 8. Most of the systematic reviews were of critically low quality defined as “more than one critical flaw with or without non-critical weakness”. No systematic reviews included a list of excluded studies with justifications for their exclusion. Three systematic reviews had insufficient methodological rigour to be included in the overview of systematic reviews due to significant methodological limitations in their study design. Detailed quality assessment using AMSTAR 2 is documented in Additional file 6: Table 9.

### Synthesis of qualitative findings

Twenty-eight factors perceived by healthcare professionals to influence prescribing decisions for chronic conditions were identified (see Table 3). Using the GRADE-CERQual, eight factors were assessed as high confidence, eight as moderate confidence, seven as low confidence and five as very low confidence. These factors were categorised into six key themes: (1) patient factors perceived to influence prescribing decisions; (2) individualised patient risk–benefit assessment—which predominantly focussed on the risks rather than the benefits when making prescribing decisions; (3) clinical inertia—defined as the failure to initiate a treatment when indicated in this context [69]—which may result from factors related to prescribers, interactions between healthcare professionals and the healthcare system; (4) medication costs—which are important in the prescribing decision-making process and go beyond direct medication costs to the prescriber; (5) shared decision-making—which is not always shared equally between patients and prescribers when making prescribing decisions; and (6) external factors influence prescribing decisions.

**Table 3:** Summary of qualitative findings

#	Summary of qualitative review findings	Confidence according to CERQual assessment	Likelihood of review representing decisions	Studies contributing to the review finding
1	Patient factors			
1.1	Age: Elderly patients are less likely to be prescribed high-risk medications; younger patients may be steered towards non-medical interventions	High	Highly likely	[43, 46, 53, 56, 60, 64, 66]
1.2	Ethnicity: Patients from ethnic minorities may be perceived to need additional resources for decision-making and overcoming barriers	Moderate	Likely	[43, 46, 60, 64]
1.3	Socioeconomic status: Lower socioeconomic status can lead to less prescribing due to lower patient awareness reduced self-advocacy, and financial resources	Low	Possible	[53, 64]
1.4	Occupational status: Fast-acting medications are preferred for patients who will be returning to work	Very low	Unclear	[53]
1.5	Geographical factors: Patients in rural areas have less medications prescribed due to distance for monitoring and follow-up appointments	Low	Possible	[46, 52, 64]
2	Individualised patient risk–benefit assessment			
2.1	Barriers to understanding: Issues such as language, literacy, visual ability, cognitive state and mental health affect prescribing due to potential communication, self-management and adherence concerns	High	Highly likely	[46, 48, 60, 61, 64]
2.2	Comorbidities: Multiple conditions complicate prescribing due to interaction risks and other priorities	Moderate	Likely	[45, 46, 56, 60, 61, 64]
2.3	Disease features: Risk–benefit assessments vary with disease duration and symptom severity	Low	Possible	[53, 60]
2.4	Frailty: Frailty influences prescribing decisions as it increases the perceived risk	Low	Possible	[46, 53]
2.5	Healthcare professional identity: Experience, speciality and healthcare setting affect risk acceptance in prescribing	High	Highly likely	[45, 49, 51, 58, 61, 64]
2.6	Objective measures: Disease severity markers and risk assessment tools guide prescribing	Low	Possible	[51, 53]
3	Clinical inertia			
3.1	Inadequate healthcare professional knowledge: Lack of training, experience and participation in research trials contribute to clinical inertia	High	Highly likely	[45, 46, 49, 52, 53, 56, 58, 61, 64, 67]
3.2	Prescriber experience: Experience impacts decision-making and resistance to new medications	Moderate	Likely	[53, 55, 56]
3.3	Professional responsibility: Ambiguity in responsibility can cause hesitation in starting medications	High	Highly likely	[45, 46, 51, 52, 56, 58, 64, 66, 67]
3.4	Healthcare system factors: Time constraints, patient load and competing priorities contribute to clinical inertia	Moderate	Likely	[52, 53, 58]
3.5	Colleagues of the same profession: Prescribers value their colleagues’ opinions and support when making prescribing decisions, this may reduce clinical inertia	Very low	Unclear	[53]
4	Medication costs			
4.1	Medication costs: Costs influence prescribing; justification is based on patient benefit and affordability	High	Highly likely	[48, 51, 53, 64, 66]
4.2	Financial incentives: Financial incentives or reimbursement can encourage prescription of certain medications	Low	Possible	[46, 52, 66]
4.3	Healthcare system costs: Medications with an increased workload due to monitoring and follow-up appointments are less likely to be prescribed	Low	Possible	[49, 64]
4.4	Patient affordability: Prescribers consider medication costs to prevent financial harm	High	Highly likely	[46, 48, 51–53, 64]
5	Shared decision-making			
5.1	Adherence: Healthcare professionals may not offer medications due to beliefs about patient adherence	Moderate	Likely	[45, 58, 64, 67]
5.2	Assumptions about patient preferences: Assumptions can lead to unequal patient involvement in decisions	High	Highly likely	[45, 46, 51–53, 64, 66, 67]
5.3	Active requests: Patient requests can influence prescribing, benefiting those with better health literacy	Moderate	Likely	[51, 53, 60]
6	External factors			
6.1	Sales and marketing: Pharmaceutical payments can influence prescribing decisions	Very low	Unclear	[62]
6.2	Guidelines availability: Availability of guidelines positively affects prescribing	Moderate	Likely	[46, 48, 64, 67]
6.3	Guidelines relevance: Guidelines must align with local demographics and priorities to be effective	Moderate	Likely	[52, 56, 60, 64]
6.4	Academia: Clinical research findings can support decision-making but their source must be perceived to be trustworthy	Very low	Unclear	[66]
6.5	Expert opinion: The opinions of experts within a field may influence less experienced healthcare professionals’ decision-making	Very low	Unclear	[51]

#### Patient factors perceived to influence prescribing decisions

Patient-level factors were reported to shape prescribing decisions. Healthcare professionals described that an older patient age may influence their prescribing decisions [47, 54, 65, 68] due to health professionals’ considering the potential interactions of a long-term medication with other medications, weighing the risks of polypharmacy alongside possible effect on comorbidities or frailty [43, 46, 53, 56, 60, 64, 66]. When considering patients living in rural or remote areas, prescribers were concerned about barriers to follow-up appointments, monitoring or emergency appointments, which were perceived as an additional risk [53, 60, 64]. Some prescribers reported that a perceived need for additional resources for patients who could not speak the dominant language within the country or had low levels of literacy could lead to not prescribing some medications [43, 46, 60, 64]. Prescribers indicated that occupational status of patients might play a role in their decisions, with more aggressive or faster-acting medications favoured for those in employment to facilitate a quicker return to work [53]. Prescribers’ presumptions about patients’ socioeconomic status were also reported as a potential influence on prescribing decisions, with some prescribers opting not to prescribe medications if they believed the patient could not afford it [53, 64].

#### Individualised patient risk–benefit assessment predominantly focussed on the risks rather than the benefits when making prescribing decisions

When prescribing for chronic conditions, prescribers may prioritise potential risks over potential benefits [46]. They described that any potential for poor patient understanding would lead them to perceive prescribing as higher risk; this might be due to language barriers [45, 46, 48], literacy levels [46], visual impairment [46], cognitive state [60, 61, 64] or mental health [60]. Concerns about potential misunderstandings, adherence and self-management led some prescribers to decide against prescribing to avoid risks. Support from family, community health or social care was described by prescribers to give some reassurance that a medication would be used correctly, leading some prescribers to initiate a medication [46, 48, 64, 66]. Similarly the need for regular monitoring or follow-up appointments was described as reassuring by prescribers as any adverse effects or adherence issues could be detected and managed promptly, making them more likely to prescribe certain medications [49, 64].

Prescribers stated that multiple comorbidities complicated their prescribing decision-making as interactions with other medical conditions and competing priorities made risk assessment more challenging [53, 56, 60, 61, 64]. They perceived comorbidities that cause dependency or reduced coping ability as carrying the most risk, influencing the likelihood of initiating a long-term medication [53, 56, 60, 61, 64].

Patient age was reported as a common factor in risk–benefit assessments. Some prescribers preferred to offer lifestyle changes before medications to younger patients, believing that this age group might prefer to avoid taking medications long-term [37]. They reported viewing patients with longer disease durations or more severe symptoms to have more to gain from long-term medications [53, 60], but if the condition persisted for too long or led to frailty, the benefits might not outweigh the risks [53]. Prescribers described their assessment of patient frailty being based on clinical judgement and frailty assessment tools [46, 53].

More experienced healthcare professionals described being more accepting of risk when prescribing long-term medications [45, 49, 61, 64]. This experience was described as the number of years in practice but also their familiarity with the medication or regular use in clinical practice [61]. Prescriber type could influence comfortability with prescribing; some primary care practitioners described being less comfortable with risk, preferring to defer high-risk prescribing decisions [45, 51, 58, 64] or co-managing with specialists in secondary care [51]. Prescribers stated that while they primarily relied on their clinical judgement, they were also influenced by objective measures such as disease severity markers [45, 53] and risk assessment tools [51].

#### Clinical inertia—which may result from factors related to prescribers, interactions between healthcare professionals and the healthcare system

Prescribers expressed that their lack of knowledge could contribute to delays or reluctance in initiating some medications [45, 46, 52, 53, 58, 61, 67]. Potential reasons cited for this were insufficient training [49, 53, 61], limited experience [49, 53, 61], limited participation in research trials [53] or being unaware of recent advancements in the field [52, 56, 64]. More experienced or senior prescribers described making decisions in a more efficient manner but acknowledged that they might also be more resistant to change [53, 55]. Some prescribers reported putting greater trust in their personal clinical experience than in new research findings [56]. Prescribers also reported that colleagues’ opinions and support may inform their prescribing decisions [53].

Primary care practitioners described uncertainty over which medications fell within their prescribing remit [56, 67], with some perceiving some medications as not their responsibility [46, 52, 64, 66] or not concordant with their professional role or identity [45, 49, 51, 58, 61, 64]. They also report feeling less confident in high-risk or complex scenarios so defer to specialists in secondary care [45, 51, 58, 64]. Both primary and secondary care prescribers noted that communication channels between them were not necessarily well coordinated or timely which could influence their decision about whether to prescribe a medication or lead to delays [51, 52, 56, 64].

Prescribers described limited consultation time and competing priorities as additional barriers. The reasons for this included that the initiation of medications often needs time-intensive patient education, discussion of treatment options and shared decision-making [52, 53, 58]. Large numbers of patients in clinics and prescribers’ feelings of professional burnout could make the initiation of long-term medications less of a priority [52, 58], sometimes leading to a focus on acute symptom management rather than the initiation of long-term preventative medications [52, 66].

#### Medication costs—which are important in the prescribing decision-making process and go beyond direct medication costs to the prescriber

Healthcare professionals reported that medication costs were a factor when making prescribing decisions about long-term medications [48, 51, 53, 64, 66]. They described weighing these costs in their risk–benefit assessments justifying the costs when a patient was likely to highly benefit from the treatment or faced a high risk of disease progression without it [51, 53]. Although financial incentives or reimbursement might encourage them to prescribe certain medications [52], some prescribers felt uncomfortable and did not think these approaches were ethical if they did not align with best evidence practices or the medications were not relevant for their patient population [46, 66]. In healthcare settings where care is not free at the point of access, prescribers reported factoring in medications costs to patients to protect them from financial harm, though these decisions were based on their perceptions rather than direct empirical knowledge of their patients’ financial circumstances [46, 51, 53, 64]. Prescribers described that while having monitoring for medications gave them some reassurance, they might not prescribe a medication if this monitoring resulted in an increased workload, resource use or need for support staff [49, 64].

#### Shared decision-making—which is not always shared equally between patients and prescribers when making prescribing decisions

Prescribers described how shared decision-making played a role in whether they initiated long-term medications. They reported sometimes choosing not to discuss long-term medications if they thought that a patient might not want to take the medication for a range of potential reasons. These may include anticipated refusal [45, 58, 64, 67], potential difficulties with adherence [45, 58, 64, 67], reluctance to initiate new medications [52], desire to avoid polypharmacy [52] or certain side effects [53, 66], or avoidance of certain treatment modalities such as injections [45, 46, 53, 67] and interventions needing high levels of monitoring [64]. Additionally, some prescribers described not discussing certain treatments with patients, fearing it might heighten patient health anxiety if it is viewed as a “last resort” by reinforcing concerns about disease progression or death [51, 53]. When patients actively request a medication, prescribers reported they were more likely to initiate it [51]. They did recognise that this often favoured people from higher socioeconomic backgrounds who often are more literate and able to self-advocate, thus exacerbating health inequalities [53, 60].

#### External factors influence prescribing decisions

The role of sales and marketing in influencing prescribing decisions was only identified in one systematic review in which prescribers reported that payments from pharmaceutical companies to prescribers in the oncology and haematology fields made them more likely to initiate certain medications [62]. Other external factors were more commonly mentioned. Prescribers described guidelines positively influencing their decision-making to prescribe a long-term medication [46, 48, 52, 64]. However, they emphasised for guidelines to be influential they need to be developed using evidence from studies with participants that were similar to their local patient populations [44, 52, 56, 60, 64] and take into account any local psychosocial factors [64] or public health issues and priorities [44]. Any ambiguity or uncertainty in the guideline recommendations could make their prescribing decision-making more complicated so could lead to them not using the guidelines [66]. Another aspect that was mentioned by prescribers was that their use of guidelines in their decision-making depends on their accessibility in the moment [46, 48, 64] because they can find it difficult to recall recommendations [67]. Academia in the form of published research was described as influencing prescribing practices [51, 66] but some prescribers described having mistrust in this source of information [66]. Expert opinion from colleagues could also guide and support some prescribers in their decision-making [51] but the identity of these experts was not described.

### To what extent does qualitative data from chronic conditions support the existing prescribing model?

The relevance of the prescribing model proposed by Murshid and Mohaidin to prescribing decisions for chronic conditions was assessed by comparing its factors with the findings from the qualitative research in the systematic reviews. New factors and connections between these factors possibly influencing decision-making for chronic conditions were identified from the overview of systematic reviews. Some terms were changed: “physician” was replaced with “prescriber” for broader applicability, and “drug” with “medication” to avoid negative connotations and align with contemporary clinical language.

Three new factor categories were added from our systematic reviews: prescriber, healthcare system and external organisations. In addition to physician habit persistence suggested by Murshid and Mohaidin in their conceptual model, we identified other prescriber characteristics that may possibly influence prescribing decisions including the prescribers’ care setting, medical speciality, knowledge and experience, attitudes and beliefs, and approach to risk. The wider healthcare system may also influence prescribing decisions and may possibly lead to clinical inertia due to competing care priorities, time constraints, staff availability and the volume of patients in clinics. Clinical guidelines, expert opinion and academia were added to the conceptual model as external organisations identified as possibly influencing prescribing decisions for chronic conditions.

The conceptual model by Murshid and Mohaidin included two factors within the patient characteristics category: patient expectations and patient requests for medication. In addition to these, the findings from the included systematic reviews introduced further factors that may influence prescribers in the context of chronic conditions: patient demographics (age, socioeconomic status, occupation, geography, ethnicity and barriers to understanding), comorbidities (including frailty) and patient behaviours (adherence and attendance).

Additional contextual factors were identified to potentially moderate the factor categories influencing prescribing practices. These included condition factors (including disease severity, duration of disease and objective markers), risk–benefit assessment and shared decision-making. Some aspects of the condition were found to influence the likelihood of prescription, in particular disease severity and duration. Costs were also identified in the included systematic reviews but possible influences beyond the cost of the medication to the healthcare system were suggested—cost to patient and indirect costs such as monitoring or support staff.

Some factors in the Murshid and Mohaidin conceptual model were less prominent in our overview of systematic review. The pharmacist was not reported to be an influencing factor in the included systematic reviews and similarly trust between prescribers and pharmacists was not mentioned. Sales and marketing factors were infrequently mentioned by prescribers in the included systematic reviews, with only one review explicitly discussing the potential impact of pharmaceutical industry payments on prescribing decisions for haematological or ontological chronic conditions [62].

**Fig. 3:**
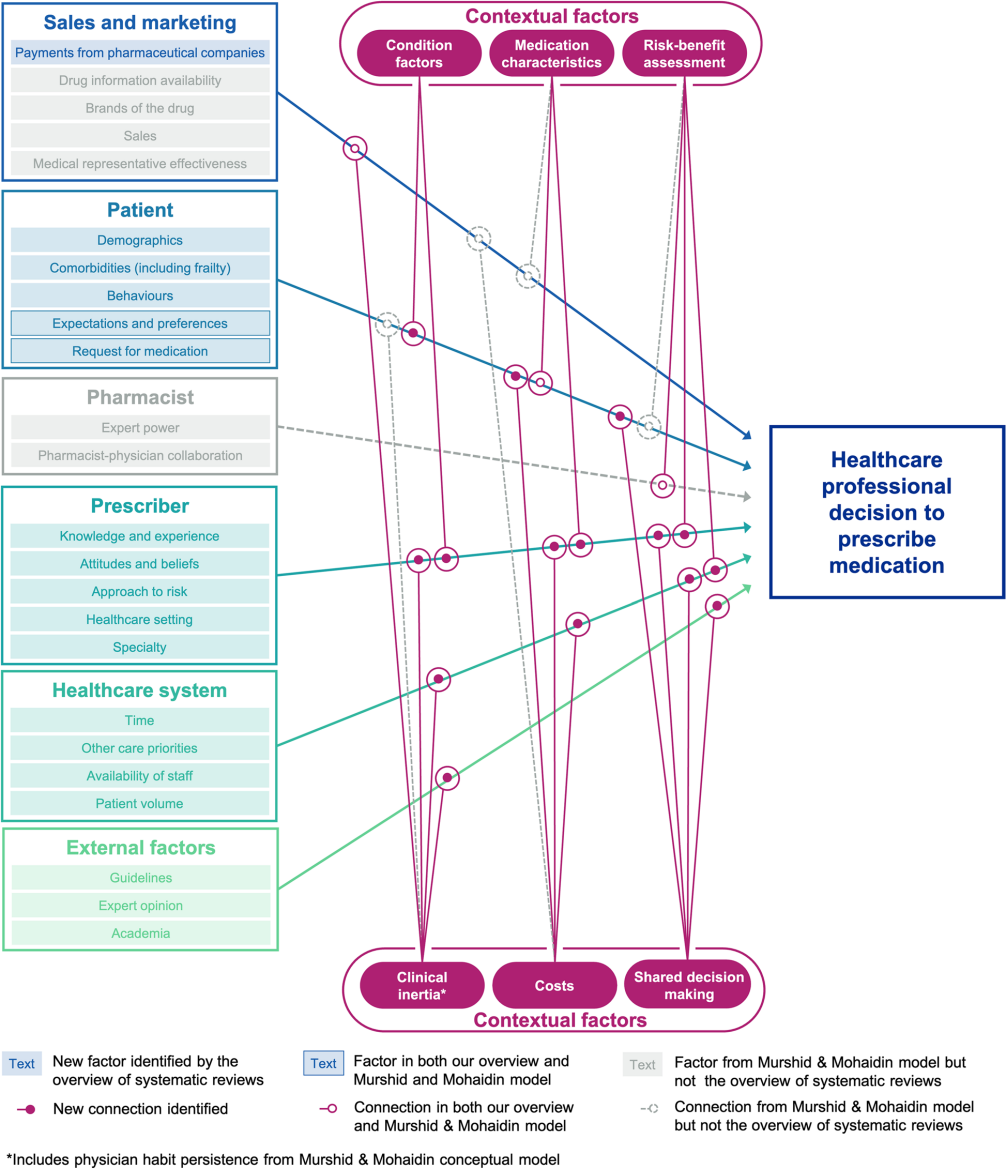
Adapted conceptual model of healthcare professional decision-making for chronic conditions

These changes are reflected in the adapted infographic (Fig. 3).

### How do factors identified in systematic reviews with qualitative findings compare to those identified in systematic reviews with quantitative findings?

Factors tested in the quantitative research were categorised into the themes identified in the qualitative research (Table 4). Many of the factors described in the systematic reviews of qualitative research were not tested in the systematic reviews of quantitative research, particularly within the themes of medication costs, shared decision-making and external factors. The factors tested were primarily related to patient characteristics. Patients who were of older age [47, 54, 65, 68], from minority ethnic backgrounds [43], living in rural areas [44, 65], multimorbid [44, 47, 54, 68] or frail [59, 63, 65, 68] were less likely to be prescribed certain medications. Evidence of higher disease severity of activity through objective scores was associated with an increased prescribing rate [50, 54, 57]. At the prescriber level, a higher self-reported level of knowledge [44, 57], participation in specialist training [50] and certain specialisms [50] were associated with higher rates of prescribing.

**Table 4:** Summary of quantitative factors in relation to qualitative findings

Qualitative findings	Quantitative factor	Observed association with prescribing	Study
**1 Patient factors**			
1.1 Age	Older patient age	Lower prescribing rate observed	[47, 54, 65, 68]
1.2 Ethnicity	Patient ethnicity	Inconsistent observations	[43]
1.3 Socioeconomic status	NA	NA	NA
1.4 Occupational status	NA	NA	NA
1.5 Geographical factors	Patients living in rural area	Lower prescribing rate observed	[44, 65]
NA	Female	Mixed	[47, 50, 54]
NA	High educational attainment	Higher prescribing rates observed	[60]
NA	Overweight or high BMI	Inconsistent observations	[54]
**2 Individualised risk–benefit assessment**			
2.1 Barriers to understanding	NA	NA	NA
2.2 Comorbidities	Multi-comorbid	Lower prescribing rate observed	[44, 47, 54, 68]
2.3 Disease features	NA	NA	NA
2.4 Frailty	Nursing home residency or frailty	Lower prescribing rate observed	[59, 63, 65, 68]
2.5 Healthcare professional identity	NA	NA	NA
2.6 Objective measures	High markers of disease activity	Higher prescribing rate observed	[50, 54, 57]
**3 Clinical inertia**			
3.1 Inadequate healthcare professional knowledge	Prescribers’ lack of knowledge	Lower prescribing rate observed	[44, 57]
3.2 Prescriber experience	More specialised training	Higher prescribing rate observed	[50]
	Certain specialities	Inconsistent observations	[50]
3.3 Professional responsibility	NA	NA	NA
3.4 Healthcare system factors	NA	NA	NA
3.5 Colleagues of the same profession	NA	NA	NA
NA	Pharmacist involvement	Higher prescribing rate observed	[57]
**4 Medication costs**			
4.1 Medication costs	NA	NA	NA
4.2 Financial incentives	NA	NA	NA
4.3 Healthcare system costs	NA	NA	NA
4.4 Patient affordability	NA	NA	NA
**5 Shared decision-making**			
5.1 Adherence	NA	NA	NA
5.2 Assumptions about patient preferences	NA	NA	NA
5.3 Active requests	NA	NA	NA
**6 External factors**			
6.1 Sales and marketing	NA	NA	NA
6.2 Guidelines availability	NA	NA	NA
6.3 Guidelines relevance	NA	NA	NA
6.4 Academia	NA	NA	NA
6.5 Expert opinion	NA	NA	NA
NA	Membership to medical society	Inconsistent observations	[50]
**Other**			
NA	Different countries	Inconsistent observations	[50]

Some factors were measured in the quantitative research that had not been identified in the qualitative research. These associations with prescribing rates were mainly at the patient level. Patients who had a higher educational attainment were associated with an increased likelihood of prescription [60]. There was no consistent direction of effect across the included systematic reviews for patients who were female [47, 50, 54] or overweight/obese [54]. For the external factors, the authors in one study linked membership to a specific medical society to an increased likelihood of prescribing a certain medication [50]. Variation in prescribing approaches for certain medications was also associated with the prescribers’ country of origin, although no consistent direction of effect was observed [50]. The influence of pharmacists in the decision-making process was not identified in any systematic reviews of qualitative research but was found to be positively associated with an increased likelihood of appropriate prescribing in one systematic review of quantitative research [57].

## Discussion

### Key findings

Twenty-six reviews published between 2013 and 2023 were included, synthesising 689 primary studies. Patient factors perceived to influence prescribers’ decisions included age, ethnicity, education and level of rurality of residence. Prescribers describe assessing individual patient characteristics when weighing the risks and benefits, with a tendency to prioritise risks—especially for patients with multiple comorbidities or complex needs. Prescribers’ approach to risk may be influenced by their clinical experience, the care setting and assessment tools. High workload and competing priorities may lead to clinical inertia in terms of delaying or preventing medication initiation. Shared decision-making may not always be shared equally between patients and prescribers. Beyond direct medication costs, prescribers may also consider broader healthcare costs, such as the need for monitoring and use of support staff for monitoring. External factors such as guidelines may be helpful in navigating risks, with their effectiveness potentially enhanced when they offer specific recommendations tailored to prescribers’ population characteristics.

### Our findings in the wider context

Our findings align with broader research from primary studies and highlight differences in prescribing decision-making from acute conditions, which is more widely studied despite being less frequent [70–73]. In acute prescribing, medications may be prescribed driven by clear benefits and immediate clinical risk of no treatment. In contrast, prescribing for chronic conditions may focus more on preventing disease progression and long-term complications [74]. The benefits of these medications can be less immediate than for acute conditions and may be harder to assess, leading healthcare professionals to focus more on potential risks. Risk assessments can also be considered more complex being shaped by factors like multiple comorbidities [75], polypharmacy [76], consequences of non-compliance [74, 77], treatment inadequacy [74] and cumulative end-organ risk [78]. While caution is needed, this heightened focus on risk may lead to overly cautious decision-making. Given that medication risks in chronic conditions may be long-term and dynamic, repeat assessments may be needed as conditions change or new medications are introduced [79].

Time constraints impact prescribing differently in acute versus chronic prescribing conditions. In acute conditions like infections, time pressures can increase the likelihood of prescribing, as some healthcare professionals prefer to avoid lengthy explanations about why antibiotics are unnecessary and challenging consultations with patients. These shorter consultations then allow them to see more patients [70–72, 80]. However, in chronic conditions, this overview of systematic reviews suggests that long-term medications may not be prescribed even when indicated, clinical inertia, when there are time pressures. This might be because long-term medications will often be taken for a longer time and may require time-consuming and complex decision-making.

Several prescriber factors may also lead to clinical inertia, including insufficient training, limited involvement in research, lack of familiarity with guidelines and a lack of awareness about recent advancements in treatment options [81–84]. When prescribing for chronic conditions, there may be a lack of immediate feedback on whether the medication is having the desired effect, unlike acute conditions where the outcome is more apparent as the condition resolves quickly or symptoms are better controlled. Without clear, short-term evidence of success, prescribers may see prescribing long-term medications for chronic conditions to be lower priority especially if they are unaware of the evidence base for the medication or less experienced [85]. More experienced healthcare professionals may also experience inertia, finding it difficult to adopt new practices or unlearn outdated ones [81, 86–88] due to confidence in their clinical judgement and past experiences [89].

Prescribing inertia may manifest differently across different care settings. Primary care prescribers, managing a wide range of conditions, tend to prioritise risk reduction and adhere strictly to guidelines, often hesitating to prescribe high-risk medications without specialist input due to concerns about complications [90, 91]. Communication challenges at the primary-secondary care interface further exacerbate this caution with primary care prescribers feeling they cannot easily contact specialists for support [92–95]. In contrast, secondary care prescribers are reported to be more accustomed to interpreting guidelines and calculated risks for long-term benefits [96]. However, they may defer medication initiation to primary care due to logistical issues or lack of detailed patient knowledge [97, 98]. Unclear responsibility between primary and secondary care may lead to clinical inertia, as neither setting feels fully accountable for initiating treatment [99], with efforts like prescribing agreements to address this [97]. Despite these challenges, some scholars report that primary care adopts approaches from secondary care, though with differing levels of specialised knowledge, varying degrees of responsibility and occasional increases in error rates [18, 100–102].

The overview did not include many systematic reviews that described the impact of sales and marketing or the pharmaceutical industry on prescribing in chronic conditions. Other reviews and primary studies have demonstrated mixed findings ranging from no influence [18, 103–106] to significant positive influence [107–110]. Thus, their absence does not mean that these factors do not affect prescribing decisions for long-term medications in chronic conditions. A potential explanation for the lack of detailed inclusion in our overview may be the tendency of studies assessing sales and marketing impact to focus on new therapeutic agents or single medications, which may not generate sufficient evidence to inform systematic reviews [111]. Additionally, these factors can be difficult for prescribers to raise because they may perceive them to be external to their decision-making, be concerned about potential conflicts of interest, or hesitate—consciously or unconsciously—to reveal their true views or beliefs about whether their decisions are influenced [107].

Guidelines featured as a factor perceived to influence prescribing practices, despite not being included in the conceptual model by Murshid and Mohaidin. There are some considerations for guidelines as they may only influence decision-making if prescribers believe they adequately represent their patient population. This may be limited by clinical trial recruitment as they are not always reflective of real-world patients [112, 113]. Although not found in our overview, there are other external organisations that may influence prescribers, such as patient advocacy groups [18, 114], leading experts [115], public opinion [18] and media sources [18]. Clinical decision support systems may also influence prescribing decisions with the aim to make prescription decisions faster, more accessible and easier [116].

The factors identified in the systematic reviews with quantitative findings were predominantly patient-level factors. This is not unsurprising as these factors are relatively easy to measure and report. Although these factors are largely non-modifiable, their consideration remains important as assumptions prescribers hold about patients with these characteristics may be incorrect and these assumptions may be modifiable. Thus, interventions at the policy or guideline levels could address this potential source of unequal patient access to medications.

A reflection on the evidence base for our overview is that, despite aiming to include all prescriber types, the studies primarily focussed on physicians, with little mention of other clinical disciplines [117, 118]. This gap may reflect the dominance of physicians in both research and clinical prescribing practice [117]. Alternatively, it could be due to nurses and allied health prescribers being more involved in acute care, prescribing antibiotics and analgesics, rather than managing chronic conditions [11, 119]. While our overview did not identify any systematic reviews on allied health prescribers in chronic condition management, there are systematic reviews and primary studies on generic prescribing that highlight differences between allied health prescribers and their physician colleagues. Pharmacists are described as being potentially more influenced by guidelines, peer recommendations and costs than their physician counterparts [18, 110, 120]. Prescribing decisions among nurses may be differentially influenced by the degree of role clarity [117] and the level of support they receive from physician colleagues [11, 117]. Additionally, nurses may be more concerned about potential risks [11, 117] and more susceptible to competing care priorities compared to physicians [11, 117, 119].

#### Strengths and weaknesses

A key strength of our review is the inclusion of both qualitative and quantitative evidence, offering a more comprehensive view of factors that may influence prescribing decisions. By incorporating perspectives from both primary and secondary care, our findings are more generalisable across the healthcare system. Additionally, the use of inclusive terminology in the conceptual model for prescribing in chronic conditions facilitates applicability to a variety of prescribers. Focussing on chronic condition management, where most prescribing occurs and decision-making is particularly complex, addresses a gap in the literature.

Our overview has some limitations. First, the inclusion of studies with diverse designs, populations and disease areas introduced heterogeneity, complicating data synthesis. Nevertheless, this heterogeneity makes the findings more transferable to chronic conditions as a whole. Second, although the aim was to include prescribers from all backgrounds, the perspectives of allied health prescribers were not fully explored in the included systematic reviews, limiting the transferability of our findings to this type of prescriber and highlighting an area for future research. Finally, the studies included in the overview of systematic reviews were predominantly from high-income countries so our findings may not represent the factors that may influence prescribing in lower-income countries.

#### Implications for clinical practice and areas for future research

The findings have four implications for clinical practice. First, prescribers must be mindful of potential biases, such as assumptions about patient adherence or ability to pay for medications, to reduce the potential for care inequalities. Second, more balanced risk–benefit assessments may be needed, considering not only the risks but also the potential benefits of medications, particularly for older or frail patients. There could be a greater emphasis on shared decision-making and improved communication of risk–benefit information with patients. Third, clinicians’ experience and comfort with risk shapes their decisions, highlighting the need for additional training and decision support tools. Fourth, clinical inertia driven by time constraints, communication gaps at the primary-secondary care interface and competing priorities underscores the need for better coordination and clearer delineation of prescribing responsibilities.

Future research should distinguish prescribing in chronic and acute conditions, as different factors may influence decision-making. While guidelines can positively influence prescribing, the clinical trials on which guidelines are based may benefit from including a diverse range of participants to reflect real-world populations. The limited inclusion of pharmacists and sales and marketing factors in our conceptual model of prescribing in chronic conditions suggests these factors may be more difficult to identify using qualitative research and thus are under-reported. Significant knowledge gaps exist around the prescribing decisions of allied health prescribers in chronic care, highlighting the need for targeted research to further explore prescribing practices across these clinical roles.

## Conclusions

The conceptual model presented, based on an overview of systematic reviews, captures the complexities of prescribing decisions in the context of chronic conditions. Unlike existing models—such as that proposed by Murshid and Mohaidin, which address prescribing more generally—this model is specifically tailored to the challenges of long-term medications for chronic conditions. It suggests possible interrelated factor categories influencing prescribing. The conceptual model may be used as a framework for future research and the development of interventions related to the factors influencing prescribing decisions in chronic conditions.

## Supplementary Information

Additional file 1: Table 1 List of chronic diseases.

Additional file 2: Tables 2–6. Table 2 MEDLINEsearch strategy. Table 3 EMBASEsearch strategy. Table 4 Web of Science search strategy. Table 5 Cochrane Library search strategy. Table 6 Google Scholar search strategy.

Additional file 3: Fig. 1 Detailed PRISMA diagram.

Additional file 4: Table 7 Detailed summary of included studies.

Additional file 5: Table 8 Excluded studies and reasons at full-text screening.

Additional file 6: Table 9 Detailed AMSTAR 2 quality assessment.

## Data Availability

All data analysed in this study are derived from previously published systematic reviews, which are fully cited in the reference list. Extracted data – including search strategies, study characteristics, and quality assessments – are provided in Additional files 2–6.

## Abbreviations

PRISMA: Preferred reporting items for systematic reviews and meta-analyses
ENTREQ: Enhancing transparency in reporting the synthesis of qualitative research
PROSPERO: International prospective register of systematic reviews
SPIDER: Sample, Phenomenon of Interest, Design, Evaluation and Research type
AMSTAR 2: Assessment of multiple systematic reviews-2
GRADE-CERQual: Grading of recommendations assessment, development and evaluation—confidence in evidence from reviews of qualitative research
NICE: National Institute for Health and Care Excellence
GMC: General Medical Council
NHS: National Health Services
UK: United Kingdom
US: United States
