# Supplementary material for: A conceptual model of factors potentially influencing prescribing decisions for chronic conditions: an overview of systematic reviews
Source: BMC Med. 2025 Jul 1;23:364. doi: 10.1186/s12916-025-04194-9 (PMC12217990; doi:10.1186/s12916-025-04194-9)
Supplement: Supplementary file 1 — Additional file 1: Table 1 List of chronic diseases. [file 12916_2025_4194_MOESM1_ESM.docx]

## Table 1. List of chronic diseases

| **#** | **Chronic Disease** |
| --- | --- |
| 1 | Addictions – substance and alcohol, etc. |
| 2 | ADHD |
| 3 | Allergic rhinitis/rhinitis/sinusitis/rhinosinusitis |
| 4 | Allergy/anaphylaxis |
| 5 | Amnesia |
| 6 | Amputations |
| 7 | Anaemia |
| 8 | Angina |
| 9 | Angiooedema |
| 10 | Anklosing spondylitis (and other arthritic conditions) |
| 11 | Antenatal screening for haemoglobinoapthies – sickle cell and thalassemia, Downs |
| 12 | Anxiety and stress disorders (including complex and post-traumatic stress disorders) |
| 13 | Aphasia |
| 14 | Asthma |
| 15 | Ataxia’s |
| 16 | Atrial fibrillation |
| 17 | Autism |
| 18 | Autoimmune disorders (e.g. lupus, Sjögrens syndrome) |
| 19 | Blood disorders |
| 20 | Brain injuries (including stroke and TIAs) |
| 21 | Bronchopulmonary dysplasia (chronic lung disease of infancy) |
| 22 | Burn injuries |
| 23 | Cancer |
| 24 | Cardiac arrhythmias |
| 25 | Cerebral palsy |
| 26 | Childhood constipation |
| 27 | Chronic fatigue syndrome/ME |
| 28 | Chronic kidney disease |
| 29 | Chronic obstructive pulmonary disease |
| 30 | Chronic pain |
| 31 | Coeliac disease |
| 32 | Congestive heart failure |
| 33 | Connective tissue diseases |
| 34 | Coronary heart disease |
| 35 | Crohn’s disease |
| 36 | Cystic Fibrosis |
| 37 | Dementia |
| 38 | Depression |
| 39 | Diabetes: Type I |
| 40 | Diabetes: Type II |
| 41 | Digestive conditions, stomach ulcers, oesophagus, reflux |
| 42 | Dizziness |
| 43 | Dyslexia or dyspaxia |
| 44 | Eating disorders (anorexia/bulimia) |
| 45 | Eczema |
| 46 | Endocrine disorders (thyrotoxicosis, hypothyroidism, hypogonadism, Cushing syndrome, Addison’s disease) |
| 47 | Endometriosis |
| 48 | Epilepsy |
| 49 | Fibromyalgia/chronic widespread pain |
| 50 | Gout |
| 51 | Gynaecological problems, chronic pelvic pain |
| 52 | Haemophilia and other coagulation disorders |
| 53 | Heart failure |
| 54 | Hepatitis B |
| 55 | Hepatitis C |
| 56 | HIV |
| 57 | Hypertension |
| 58 | Inflammatory bowel disease |
| 59 | Irritable bowel disease |
| 60 | Learning disabilities |
| 61 | Low back pain |
| 62 | Lung fibrosis |
| 63 | Lupus |
| 64 | Malaria |
| 65 | Medically unexplained symptoms |
| 66 | Migraine |
| 67 | Mood disorders (not only depression, but mania and bipolar disorders) |
| 68 | Motor neurone disease |
| 69 | Multimorbidity |
| 70 | Multiple sclerosis |
| 71 | Multisystem autoimmune diseases (MSAIDs, including lupus) |
| 72 | Muscular dystrophy(ies) |
| 73 | Neuralgias (including, head and back pain) |
| 74 | Newborn screening programme diseases, including thyroid disease, hearing loss |
| 75 | Obesity |
| 76 | Obstructive sleep apnoea |
| 77 | Occupational lung disease (various) |
| 78 | Osteoarthritis |
| 79 | Osteoporosis |
| 80 | Other slowly degenerative neurological conditions |
| 81 | Parkinson’s disease |
| 82 | Peripheral vascular disease |
| 83 | Personality disorders |
| 84 | Phobias |
| 85 | Physical disabilities |
| 86 | Polycystic ovary disease |
| 87 | Post-traumatic stress |
| 88 | Progressive supranuclear palsy |
| 89 | Psoriasis |
| 90 | Rare disease, genetic disorders |
| 91 | Sarcoidosis |
| 92 | Sensory problems/disabilities (deafness/blindness) |
| 93 | Severe skin conditions |
| 94 | Sickle cell disease |
| 95 | Skin conditions |
| 96 | Sleep disorders |
| 97 | Speech deficits |
| 98 | Spina bifida |
| 99 | Spinal injuries |
| 100 | Stroke/transient ischaemic attacks |
| 101 | Tuberculosis |
| 102 | Urinary Incontinence |
| 103 | Urticarial |
