# Supplementary material for: A conceptual model of factors potentially influencing prescribing decisions for chronic conditions: an overview of systematic reviews
Source: BMC Med. 2025 Jul 1;23:364. doi: 10.1186/s12916-025-04194-9 (PMC12217990; doi:10.1186/s12916-025-04194-9)
Supplement: Supplementary file 2 — Additional file 2: Tables 2–6. Table 2 MEDLINEsearch strategy. Table 3 EMBASEsearch strategy. Table 4 Web of Science search strategy. Table 5 Cochrane Library search strategy. Table 6 Google Scholar search strategy. [file 12916_2025_4194_MOESM2_ESM.docx]

# **Tables 2-6. Full database search strategy**

## Table 2. MEDLINE (Ovid) search strategy

| **#** | **MEDLINE (Ovid) Search Lines (searched 07/11/2023)** |
| --- | --- |
| 1 | exp rhinitis/ |
| 2 | ((vasomotor or allerg* or non-allerg* or nonallerg* or non allerg*) and rhinitis).ti,ab. |
| 3 | exp amnesia/ |
| 4 | (amnesi* or memory loss).ti,ab. |
| 5 | amputation, surgical/ |
| 6 | amputation, traumatic/ |
| 7 | (amputation or amputee*).ti,ab. |
| 8 | exp anemia/ |
| 9 | an?emia.ti,ab. |
| 10 | exp cardiovascular disease/ |
| 11 | (angina or myocardial isch?mi*).ti,ab. |
| 12 | exp angioedema/ |
| 13 | (angioedema or (hereditary adj (angioedema* or angioneurotic edema*))).ti,ab. |
| 14 | exp arthritis/ |
| 15 | ((rheumatic or rheumatoid) adj3 (arthrit* or artrit* or diseas* or condition* or nodule*)).ti,ab. |
| 16 | exp aphasia/ |
| 17 | (primary progressive aphasia or aphasia or dysphasia).ti,ab. |
| 18 | exp ataxia/ |
| 19 | ((cerebell* or spinocerebell*) adj (ataxia* or incoordination)).ti,ab. |
| 20 | exp autoimmune diseases/ |
| 21 | (addison* disease or autoimmune thyroid disease or ank* spondy* or h?emolytic an?emia or multiple sclerosis or myasthenia gravis or immune thrombocytopen* purpura or scleroderma or polyarteriti* nodosa or pernicious an?emia or vitiligo or psoriasis or primary biliary cirrhosis).ti,ab. |
| 22 | exp hematologic diseases/ |
| 23 | (leukaemi* or myeloma or lymphoma).ti,ab. |
| 24 | exp stroke/ |
| 25 | exp brain ischemia/ |
| 26 | ischemic attack, transient/ |
| 27 | (stroke or poststroke or post-stroke or cerebrovascu* or brain vasc* or cerebral vasc* or cva* or apoplexy* or sah).ti,ab. |
| 28 | ((brain* or cerebr* or vascular or cerebel* or intracran* or intracerebral* or subarachnoid) adj (accident or isch?mi or infarct* or thrombo* or emboli* or occlus* or h?morrhage or h?matoma* or bleed*)).ti,ab. |
| 29 | exp burns/ |
| 30 | (burn* or scald*).ti,ab. |
| 31 | exp neoplasms/ |
| 32 | (cancer* or oncolog* or neoplasm* or carcinom* or tumo?r* or malignan* or leuk?emia).ti,ab. |
| 33 | exp arrhythmias, cardiac/ |
| 34 | cardiac arrhythm*.ti,ab. |
| 35 | cerebral palsy/ |
| 36 | cerebral pals*.ti,ab. |
| 37 | celiac disease/ |
| 38 | (c?eliac adj (disease or sprue or syndrome)).ti,ab. |
| 39 | (gluten adj (enteropath* or sensitiv* or hypersensitiv* or intoleran*)).ti,ab. |
| 40 | exp connective tissue diseases/ |
| 41 | (connective tissue disease* or sharp syndrome or polymyositis or systemic sclerosis or scleroderma).ti,ab. |
| 42 | exp myocardial ischemia/ |
| 43 | (heart disease* or myocardial ischemia or coronary disease* or coronary artery disease* or coronary heart disease* or myocardial infarct*).ti,ab. |
| 44 | exp hypersensitivity/ |
| 45 | exp allergy/ and immunology/ |
| 46 | anaphylaxis/ |
| 47 | (allerg* or hypersensitivit* or tierg* or intolerance or anaphyla*).ti,ab. |
| 48 | exp asthma/ |
| 49 | bronchial spasm/ |
| 50 | bronchoconstriction/ |
| 51 | ((bronchial* or respiratory or airway* or lung*) adj (hypersensitive* or hyperreactiv* or allerg* or insufficiency)).ti,ab. |
| 52 | (bronch* adj3 (constrict* or spas*)).ti,ab. |
| 53 | (asthma* or wheez* or bronchoconstrict* or antiasthma* or anti-asthma* or bronchal hyperreactivity or bronchospas*).ti,ab. |
| 54 | atrial fibrillation/ |
| 55 | ((atrial or atrium) adj fibril?at*).ti,ab. |
| 56 | fatigue syndrome, chronic/ |
| 57 | exp epilepsies, myoclonic/ |
| 58 | (dravet syndrome* or severe myoclonic epilepsy of infancy or severe myoclonic epilepsy in infancy or severe polymorphic epilepsy of infancy).ti,ab. |
| 59 | exp kidney failure, chronic/ |
| 60 | (chronic adj (kidney or kidney) adj (disease* or failure or insufficien*)).ti,ab. |
| 61 | (CKD or CRF or CRD or ESKD or ESRD or ESRF).ti,ab. |
| 62 | exp pulmonary disease, chronic obstructive/ |
| 63 | (obstruct* adj (pulmonary or lung* or airway* or airflow* or bronch* or respirat*)).ti,ab. |
| 64 | (chronic* adj bronchiti*).ti,ab. |
| 65 | (chronic obstructive pulmonary disease or chronic obstructive airways disease or lung disease or emphysema* or COPD or CAL or COAD or COLD or CBD or AECB).ti,ab. |
| 66 | exp heart failure/ |
| 67 | ((heart or cardiac or myocardial) adj (failure or decompensation)).ti,ab. |
| 68 | ((congestive or acute or decompensat* or chronic) adj heart failure).ti,ab. |
| 69 | exp dementia/ |
| 70 | ((cerebr* or brain or cogniti*) adj (deteriorat* or insufficient* or disease or syndrome or impair*)).ti,ab. |
| 71 | (mild cognitive impairment or dement* or alzheimer* or binswanger* or benign senescent forgetfulness).ti,ab. |
| 72 | ((dilated or congestive) adj cardiomyopath*).ti,ab. |
| 73 | (lewy* adj bod*).ti,ab. |
| 74 | ((memory* or mental*) adj (declin* or deteriorat* or impair* or los*)).ti,ab. |
| 75 | exp diabetes mellitus/ |
| 76 | (diabet* adj3 type adj3 (one or "1" or I)).ti,ab. |
| 77 | (diabet* adj3 type adj3 (two or "2" or II)).ti,ab. |
| 78 | ((insulin or noninsulin or non-insulin) adj2 (resistan* or depend*)).ti,ab. |
| 79 | (diabet* or DM or DM1 or DM2 or T1D or T1DM or T2D or T2DM or NIDDM or IDDM or MODY).ti,ab. |
| 80 | glucose ?tolerance.ti,ab. |
| 81 | endometriosis/ |
| 82 | (endometrios* or endometrioma? or adenomyo* or adenomyoma? or adenometriti* or adenomyosit* or adenomyometriti*).ti,ab. |
| 83 | exp epilepsy/ |
| 84 | exp seizure/ |
| 85 | (epilep* or seizure* or aura* or convulsion*).ti,ab. |
| 86 | exp hypertension/ |
| 87 | (hypertens* or (blood adj pressure)).ti,ab. |
| 88 | exp hepatitis, chronic/ |
| 89 | (chronic adj hepatitis).ti,ab. |
| 90 | exp HIV/ |
| 91 | (hiv infect* or hiv disease*).ti,ab. |
| 92 | exp inflammatory bowel diseases/ |
| 93 | (crohn* disease or crohn's enteritis* or ulcerative coliti* or terminal ileitis or granulomatous coliti* or ileocoliti*).ti,ab. |
| 94 | irritable bowel syndrome/ |
| 95 | ((irritable or functional or spastic) adj1 (bowel or colon)).ti,ab. |
| 96 | (IBS or mucous colit* or gastrointestinal syndrome* or functional gastrointestinal).ti,ab. |
| 97 | exp migraine disorders/ |
| 98 | (migraine or megrim or hemicrania).ti,ab. |
| 99 | exp multiple sclerosis/ |
| 100 | (multiple sclerosis or MS or demyelinating disease*).ti,ab. |
| 101 | exp osteoarthritis/ |
| 102 | (osteoarthr* or OA).ti,ab. |
| 103 | (degenerative adj (arthritis or joint*)).ti,ab. |
| 104 | exp parkinson disease/ |
| 105 | (parkinson* disease or PD or parkinson*).ti,ab. |
| 106 | cystic fibrosis/ |
| 107 | (cystic fibrosis or CF or mucoviscidosis or (fibrocystic disease adj pancrea*)).ti,ab. |
| 108 | exp gastroesophageal reflux/ |
| 109 | ulcer/ |
| 110 | exp esophagitis/ |
| 111 | ((gastroesophageal or gastrooesophageal or gastro ?esophageal) adj reflux).ti,ab. |
| 112 | (GORD or GERD or GOR or GER).ti,ab. |
| 113 | (gastric adj3 (acid or reflux)).ti,ab. |
| 114 | (reflux adj (oesophagitis or esophagitis)).ti,ab. |
| 115 | ((peptic or gastric or duodenal or stomach or oesophageal or esophageal) and ulcer*).ti,ab. |
| 116 | dizziness/ |
| 117 | exp vertigo/ |
| 118 | (vestibular neuritis or benign paroxysmal positional or BPPV or acoustic neuroma or meniere* disease or labyrinthinitis or vestibular neuronitis or vertigo*).ti,ab. |
| 119 | exp eczema/ |
| 120 | (eczema or atopic eczema or atopic dermatitis).ti,ab. |
| 121 | exp endocrine system diseases/ |
| 122 | (thyrotoxicosis* or hyperthyroid* or hypothyroid* or hypogonad* or cushing* syndrome or cushing disease or addison syndrome or addison disease or acromegaly or congenital adkidney hyperplasi*).ti,ab. |
| 123 | fibromyalgia/ |
| 124 | (fibromyositis syndrom* or pain syndrome or fibromyalgia syndrom* or fibromyalgia* or fibrositi* or fibromyositis or rheumatism or diffuse myofascial).ti,ab. |
| 125 | exp gout/ |
| 126 | (gout or gouty arthriti* or toph* or podagra or pseudogout).ti,ab. |
| 127 | exp blood coagulation disorders/ |
| 128 | (h?emophili* or von willebrand disease or clotting factor deficiency or clotting factor disease or hypercoagulable or thrombophilia).ti,ab. |
| 129 | exp learning disabilities/ |
| 130 | (((learning or intellectual) and (disabilit* or disorder or impairment or difficulties)) or special needs).ti,ab. |
| 131 | exp pulmonary fibrosis/ |
| 132 | ((cryptogenic or idiopathic) adj fibrosing alveoliti*).ti,ab. |
| 133 | (fibrocystic pulmonary dysplasia or idiopathic pulmon* fibros*).ti,ab. |
| 134 | exp lupus erythematosus, systemic/ |
| 135 | (lupus or SLE).ti,ab. |
| 136 | exp malaria/ |
| 137 | (malaria or plasmodium).ti,ab. |
| 138 | medically unexplained symptoms/ |
| 139 | medically unexplained symptoms.ti,ab. |
| 140 | exp motor neuron disease/ |
| 141 | (motor neuron* disease* or MND).ti,ab. |
| 142 | exp muscular dystrophies/ |
| 143 | ((duchenne or pseudohypertrophic or becker or facioscapulohumeral or limb-girdle or myotonic) adj muscular dystrophy).ti,ab. |
| 144 | exp obesity/ |
| 145 | (obes* or overweight or (over adj weight) or over-weight).ti,ab. |
| 146 | exp sleep apnea, obstructive/ |
| 147 | ((sleep* or nocturnal) adj2 (apnea* or apnoea*)).ti,ab. |
| 148 | (sleep* adj2 disordered adj2 breathing).ti,ab. |
| 149 | ((sleep* or nocturnal) adj2 (hypopnea* or hypopnoea* or hypo-apnea* or hypo-apnoea* or apneic-hypopneic or apnoeic-hypopnoeic)).ti,ab. |
| 150 | (OSA or SAHS or OSAHS).ti,ab. |
| 151 | asthma, occupational/ |
| 152 | (asbestosis* or pneumoconiosis or black lung or hard metal lung disease or silicosis or talcosis).ti,ab. |
| 153 | exp osteoporosis/ |
| 154 | (osteoporo* or bone density or calcaneus).ti,ab. |
| 155 | exp neurodegenerative diseases/ |
| 156 | (amyotrophic lateral sclerosis or multiple sclerosis or huntington* disease or multiple system atrophy or prion disease).ti,ab. |
| 157 | exp peripheral vascular diseases/ |
| 158 | (peripheral arterial occlusi* or peripheral artery occlusi* or peripheral arterial isch?emi* or peripheral artery isch?emi*).ti,ab. |
| 159 | (peripheral vascular disease* or peripheral arterial disease* or peripheral artery disease*).ti,ab. |
| 160 | ((limb* or lower extremity) adj isch?emi*).ti,ab. |
| 161 | (intermittent and (occlusion or claudication)).ti,ab. |
| 162 | exp disabled persons/ |
| 163 | (physical disabilit* or spinal cord injur* or motor learning disability* or dwarfism).ti,ab. |
| 164 | polycystic ovary syndrome/ |
| 165 | (polycystic ovary syndrome or polycystic ovarian syndrome or PCOS).ti,ab. |
| 166 | supranuclear palsy, progressive/ |
| 167 | (progressive supranuclear pals* or PSP or richardson* syndrome or (steele richardson olsezewski adj (disease or syndrome)) or (steele-richardson-olsezewski adj (disease or syndrome))).ti,ab. |
| 168 | exp psoriasis/ |
| 169 | psoriasis.ti,ab. |
| 170 | rare diseases/ |
| 171 | (rare dis* or rare diagnos* or orphan dis*).ti,ab. |
| 172 | exp sarcoidosis/ |
| 173 | (sarcoid or sarcoidosis).ti,ab. |
| 174 | exp deafness/ |
| 175 | exp blindness/ |
| 176 | visually impaired persons/ |
| 177 | (hearing loss or hearing aid* or deaf* or hearing impairment or hearing disorder).ti,ab. |
| 178 | (visual impairment or visually impaired or visual disorder or vision impairment).ti,ab. |
| 179 | exp anemia, sickle cell/ |
| 180 | (((h?emoglobin s disease or hbs disease or sickling disorder) and h?emoglobin s) or ((sickle cell adj (disease* or an?emia or disorder*)) or SCD)).ti,ab. |
| 181 | exp skin diseases/ |
| 182 | exp dyssomnias/ |
| 183 | (dyssomnia or sleep disorder*).ti,ab. |
| 184 | exp speech disorders/ |
| 185 | ((communication or learning or consciousness or perceptual or speech or voice or psychomotor) adj disorder*).ti,ab. |
| 186 | spina bifida occulta/ |
| 187 | (spina bifida or myelomeningocele).ti,ab. |
| 188 | exp spinal injuries/ |
| 189 | ((spine or spinal or vertebr* or neck or cervical or lumbar or sacral or thoracic or cord or whiplash) adj2 (injur* or damag* or trauma* or fracture* or compress* or contus* or lacerat* or transect* or lesion*)).ti,ab. |
| 190 | exp tuberculosis/ |
| 191 | (tuberculosis or TB).ti,ab. |
| 192 | exp urinary incontinence/ |
| 193 | urinary bladder, overactive/ |
| 194 | (bladder* adj3 (overactiv* or incontin* or continence)).ti,ab. |
| 195 | ((stress* or mixed or urg* or urin* or overflow*) adj3 (incontinen* or continence)).ti,ab. |
| 196 | exp urticaria/ |
| 197 | urticaria*.ti,ab. |
| 198 | chronic disease/ |
| 199 | ((chronic* or long-term) adj2 (diseas* or ill* or disorder* or condition*)).ti,ab. |
| 200 | or/1-199 |
| 201 | exp Drug Prescriptions/ or exp Prescriptions/ |
| 202 | (Prescri* or Prescribing Behavio?r or Prescrib* Decision* or Prescribing Practice*).ti,ab. |
| 203 | (Initiat* or Start* or Commenc* or Induc*).ti. |
| 204 | or/201-203 |
| 205 | exp Clinical decision-making/ |
| 206 | exp Health Knowledge, Attitudes, Practice/ or exp Attitude of Health Personnel/ or exp Practice Patterns, Pharmacists'/ or exp Practice Patterns, Nurses'/ or exp Practice Patterns, Physicians'/ |
| 207 | (Factor* or Determinant* or Behavio?r* or Practice* or Preference* or Pattern* or Decision* or Decid* or Influenc* or Impact* or Judg* or Barrier* or Facilit* or Help or Hinder or Compl* or Accept or Conform* or Approv* or Adher* or Strateg*).ti. |
| 208 | or/205-208 |
| 209 | (Medline or systematic review).tw. or meta-analysis.pt. |
| 210 | 200 and 204 and 208 and 209 |
| 211 | limit 210 to yr="2013-current" |

## Table 3. EMBASE(Ovid) search strategy

| **#** | **OVID EMBASE Search Lines (search date: 07/11/2023)** |
| --- | --- |
| 1 | exp rhinitis/ |
| 2 | ((vasomotor or allerg* or non-allerg* or nonallerg* or non allerg*) and rhinitis).ti. |
| 3 | exp amnesia/ |
| 4 | (amnesi* or memory loss).ti. |
| 5 | amputation, surgical/ |
| 6 | amputation, traumatic/ |
| 7 | (amputation or amputee*).ti. |
| 8 | exp anemia/ |
| 9 | an?emia.ti. |
| 10 | exp cardiovascular disease/ |
| 11 | (angina or myocardial isch?mi*).ti. |
| 12 | exp angioedema/ |
| 13 | (angioedema or (hereditary adj (angioedema* or angioneurotic edema*))).ti. |
| 14 | exp arthritis/ |
| 15 | ((rheumatic or rheumatoid) adj3 (arthrit* or artrit* or diseas* or condition* or nodule*)).ti. |
| 16 | exp aphasia/ |
| 17 | (primary progressive aphasia or aphasia or dysphasia).ti. |
| 18 | exp ataxia/ |
| 19 | ((cerebell* or spinocerebell*) adj (ataxia* or incoordination)).ti. |
| 20 | exp autoimmune diseases/ |
| 21 | (overact* disease or autoimmune thyroid disease or ank* spondy* or h?emolytic an?emia or multiple sclerosis or myasthenia gravis or immune thrombocytopen* purpura or scleroderma or polyarteriti* nodosa or pernicious an?emia or vitiligo or psoriasis or primary biliary cirrhosis).ti. |
| 22 | exp hematologic diseases/ |
| 23 | (leukaemi* or myeloma or lymphoma).ti. |
| 24 | exp stroke/ |
| 25 | exp brain ischemia/ |
| 26 | ischemic attack, transient/ |
| 27 | (stroke or poststroke or post-stroke or cerebrovascu* or brain vasc* or cerebral vasc* or cva* or apoplexy* or sah).ti. |
| 28 | ((brain* or cerebr* or vascular or cerebel* or intracran* or intracerebral* or subarachnoid) adj (accident or isch?mi or infarct* or thrombo* or emboli* or occlus* or h?morrhage or h?matoma* or bleed*)).ti. |
| 29 | exp burns/ |
| 30 | (burn* or scald*).ti. |
| 31 | exp neoplasms/ |
| 32 | (cancer* or oncolog* or neoplasm* or carcinom* or tumo?r* or malignan* or leuk?emia).ti. |
| 33 | exp arrhythmias, cardiac/ |
| 34 | cardiac arrhythm*.ti. |
| 35 | cerebral palsy/ |
| 36 | cerebral pals*.ti. |
| 37 | celiac disease/ |
| 38 | (c?eliac adj (disease or sprue or syndrome)).ti. |
| 39 | (gluten adj (enteropath* or sensitiv* or hypersensitiv* or intoleran*)).ti. |
| 40 | exp connective tissue diseases/ |
| 41 | (connective tissue disease* or sharp syndrome or polymyositis or systemic sclerosis or scleroderma).ti. |
| 42 | exp myocardial ischemia/ |
| 43 | (heart disease* or myocardial ischemia or coronary disease* or coronary artery disease* or coronary heart disease* or myocardial infarct*).ti. |
| 44 | exp hypersensitivity/ |
| 45 | exp allergy/ and immunology/ |
| 46 | anaphylaxis/ |
| 47 | (allerg* or hypersensitiv * or tierg* or intolerance or anaphyla*).ti. |
| 48 | exp asthma/ |
| 49 | bronchial spasm/ |
| 50 | bronchoconstriction/ |
| 51 | ((bronchial* or respiratory or airway* or lung*) adj (hypersensitive* or hyperreactive* or allerg* or insufficiency)).ti. |
| 52 | (bronch* adj3 (constrict* or spas*)).ti. |
| 53 | (asthma* or wheez* or bronchoconstrict* or antiasthma* or anti-asthma* or bronchal hyperreactivity or bronchospas*).ti. |
| 54 | atrial fibrillation/ |
| 55 | ((atrial or atrium) adj fibril?at*).ti. |
| 56 | fatigue syndrome, chronic/ |
| 57 | exp epilepsies, myoclonic/ |
| 58 | (dravet syndrome* or severe myoclonic epilepsy of infancy or severe myoclonic epilepsy in infancy or severe polymorphic epilepsy of infancy).ti. |
| 59 | exp kidney failure, chronic/ |
| 60 | (chronic adj (kidney or kidney) adj (disease* or failure or insufficien*)).ti. |
| 61 | (CKD or CRF or CRD or ESKD or ESRD or ESRF).ti. |
| 62 | exp pulmonary disease, chronic obstructive/ |
| 63 | (obstruct* adj (pulmonary or lung* or airway* or airflow* or bronch* or respirat*)).ti. |
| 64 | (chronic* adj bronchiti*).ti. |
| 65 | (chronic obstructive pulmonary disease or chronic obstructive airways disease or lung disease or emphysema* or COPD or CAL or COAD or COLD or CBD or AECB).ti. |
| 66 | exp heart failure/ |
| 67 | ((heart or cardiac or myocardial) adj (failure or decompensation)).ti. |
| 68 | ((congestive or acute or decompensat* or chronic) adj heart failure).ti. |
| 69 | exp dementia/ |
| 70 | ((cerebr* or brain or cogniti*) adj (deteriorat* or insufficient* or disease or syndrome or impair*)).ti. |
| 71 | (mild cognitive impairment or dement* or alzheimer* or binswager * or benign senescent forgetfulness).ti. |
| 72 | ((dilated or congestive) adj cardiomyopath*).ti. |
| 73 | (lewy* adj bod*).ti. |
| 74 | ((memory* or mental*) adj (declin* or deteriorat* or impair* or los*)).ti. |
| 75 | exp diabetes mellitus/ |
| 76 | (diabet* adj3 type adj3 (one or “1” or I)).ti. |
| 77 | (diabet* adj3 type adj3 (two or “2” or II)).ti. |
| 78 | ((insulin or noninsulin or non-insulin) adj2 (resistan* or depend*)).ti. |
| 79 | (diabet* or DM or DM1 or DM2 or T1D or T1DM or T2D or T2DM or NIDDM or IDDM or MODY).ti. |
| 80 | glucose ?tolerance.ti. |
| 81 | endometriosis/ |
| 82 | (endometrios* or endometrioma? Or adenomyo* or adenomyoma? Or adenometriti* or adenomyosit* or adenomyometriti*).ti. |
| 83 | exp epilepsy/ |
| 84 | exp seizure/ |
| 85 | (epilep* or seizure* or aura* or convulsion*).ti. |
| 86 | exp hypertension/ |
| 87 | (hypertens* or (blood adj pressure)).ti. |
| 88 | exp hepatitis, chronic/ |
| 89 | (chronic adj hepatitis).ti. |
| 90 | exp HIV/ |
| 91 | (hiv infect* or hiv disease*).ti. |
| 92 | exp inflammatory bowel diseases/ |
| 93 | (crohn* disease or crohn’s enteritis* or ulcerative veract* or terminal ileitis or granulomatous veract* or ileocoliti*).ti. |
| 94 | irritable bowel syndrome/ |
| 95 | ((irritable or functional or spastic) adj1 (bowel or colon)).ti. |
| 96 | (IBS or mucous colit* or gastrointestinal syndrome* or functional gastrointestinal).ti. |
| 97 | exp migraine disorders/ |
| 98 | (migraine or megrim or hemicrania).ti. |
| 99 | exp multiple sclerosis/ |
| 100 | (multiple sclerosis or MS or demyelinating disease*).ti. |
| 101 | exp osteoarthritis/ |
| 102 | (osteoarthr* or OA).ti. |
| 103 | (degenerative adj (arthritis or joint*)).ti. |
| 104 | exp parkinson disease/ |
| 105 | (Parkinson* disease or PD or parkinson*).ti. |
| 106 | cystic fibrosis/ |
| 107 | (cystic fibrosis or CF or mucoviscidosis or (fibrocystic disease adj pancrea)).ti. |
| 108 | exp gastroesophageal reflux/ |
| 109 | ulcer/ |
| 110 | exp esophagitis/ |
| 111 | ((gastroesophageal or gastrooesophageal or gastro ?esophageal) adj reflux).ti. |
| 112 | (GORD or GERD or GOR or GER).ti. |
| 113 | (gastric adj3 (acid or reflux)).ti. |
| 114 | (reflux adj (oesophagitis or esophagitis)).ti. |
| 115 | ((peptic or gastric or duodenal or stomach or oesophageal or esophageal) and ulcer*).ti. |
| 116 | dizziness/ |
| 117 | exp vertigo/ |
| 118 | (vestibular neuritis or benign paroxysmal positional or BPPV or acoustic neuroma or meniere* disease or labyrinthinitis or vestibular neuronitis or vertigo*).ti. |
| 119 | exp eczema/ |
| 120 | (eczema or atopic eczema or atopic dermatitis).ti. |
| 121 | exp endocrine system diseases/ |
| 122 | (thyrotoxicosis* or hyperthyroid* or hypothyroid* or hypogonad* or cushing* syndrome or cushing disease or veract syndrome or veract disease or acromegaly or congenital adkidney hyperplasi*).ti. |
| 123 | fibromyalgia/ |
| 124 | (fibromyositis syndrom* or pain syndrome or fibromyalgia syndrom* or fibromyalgia* or fibrositi* or fibromyositis or rheumatism or diffuse myofascial).ti. |
| 125 | exp gout/ |
| 126 | (gout or gouty arthriti* or toph* or podagra or pseudogout).ti. |
| 127 | exp blood coagulation disorders/ |
| 128 | (h?emophili* or von willebrand disease or clotting factor deficiency or clotting factor disease or hypercoagulable or thrombophilia).ti. |
| 129 | exp learning disabilities/ |
| 130 | (((learning or intellectual) and (disabilit* or disorder or impairment or difficulties)) or special needs).ti. |
| 131 | exp pulmonary fibrosis/ |
| 132 | ((cryptogenic or idiopathic) adj fibrosing alveoliti*).ti. |
| 133 | (fibrocystic pulmonary dysplasia or idiopathic pulmon* fibros*).ti. |
| 134 | exp lupus erythematosus, systemic/ |
| 135 | (lupus or SLE).ti. |
| 136 | exp malaria/ |
| 137 | (malaria or plasmodium).ti. |
| 138 | medically unexplained symptoms/ |
| 139 | medically unexplained symptoms.ti. |
| 140 | exp motor neuron disease/ |
| 141 | (motor neuron* disease* or MND).ti. |
| 142 | exp muscular dystrophies/ |
| 143 | ((overacti or pseudohypertrophic or becker or facioscapulohumeral or limb-girdle or myotonic) adj muscular dystrophy).ti. |
| 144 | exp obesity/ |
| 145 | (obes* or overweight or (over adj weight) or over-weight).ti. |
| 146 | exp sleep apnea, obstructive/ |
| 147 | ((sleep* or nocturnal) adj2 (apnea* or apnoea*)).ti. |
| 148 | (sleep* adj2 disordered adj2 breathing).ti. |
| 149 | ((sleep* or nocturnal) adj2 (hypopnea* or hypopnoea* or hypo-apnea* or hypo-apnoea* or apneic-hypopneic or apnoeic-hypopnoeic)).ti. |
| 150 | (OSA or SAHS or OSAHS).ti. |
| 151 | asthma, occupational/ |
| 152 | (asbestosis* or pneumoconiosis or black lung or hard metal lung disease or silicosis or talcosis).ti. |
| 153 | exp osteoporosis/ |
| 154 | (osteoporo* or bone density or calcaneus).ti. |
| 155 | exp neurodegenerative diseases/ |
| 156 | (amyotrophic lateral sclerosis or multiple sclerosis or huntington* disease or multiple system atrophy or prion disease).ti. |
| 157 | exp peripheral vascular diseases/ |
| 158 | (peripheral arterial occlusi* or peripheral artery occlusi* or peripheral arterial isch?emi* or peripheral artery isch?emi*).ti. |
| 159 | (peripheral vascular disease* or peripheral arterial disease* or peripheral artery disease*).ti. |
| 160 | ((limb* or lower extremity) adj isch?emi*).ti. |
| 161 | (intermittent and (occlusion or claudication)).ti. |
| 162 | exp disabled persons/ |
| 163 | (physical disabilit* or spinal cord injur* or motor learning disability* or dwarfism).ti. |
| 164 | polycystic ovary syndrome/ |
| 165 | (polycystic ovary syndrome or polycystic ovarian syndrome or PCOS).ti. |
| 166 | supranuclear palsy, progressive/ |
| 167 | (progressive supranuclear pals* or PSP or richardson* syndrome or (steele richardson olsezewski adj (disease or syndrome)) or (steele-richardson-olsezewski adj (disease or syndrome))).ti. |
| 168 | exp psoriasis/ |
| 169 | psoriasis.ti. |
| 170 | rare diseases/ |
| 171 | (rare dis* or rare diagnos* or orphan dis*).ti. |
| 172 | exp sarcoidosis/ |
| 173 | (sarcoid or sarcoidosis).ti. |
| 174 | exp deafness/ |
| 175 | exp blindness/ |
| 176 | visually impaired persons/ |
| 177 | (hearing loss or hearing aid* or deaf* or hearing impairment or hearing disorder).ti. |
| 178 | (visual impairment or visually impaired or visual disorder or vision impairment).ti. |
| 179 | exp anemia, sickle cell/ |
| 180 | (((h?emoglobin s disease or hbs disease or sickling disorder) and h?emoglobin s) or ((sickle cell adj (disease* or an?emia or disorder*)) or SCD)).ti. |
| 181 | exp skin diseases/ |
| 182 | exp dyssomnias/ |
| 183 | (dyssomnia or sleep disorder*).ti. |
| 184 | exp speech disorders/ |
| 185 | ((communication or learning or consciousness or perceptual or speech or voice or psychomotor) adj disorder*).ti. |
| 186 | spina bifida occulta/ |
| 187 | (spina bifida or myelomeningocele).ti. |
| 188 | exp spinal injuries/ |
| 189 | ((spine or spinal or vertebr* or neck or cervical or lumbar or sacral or thoracic or cord or whiplash) adj2 (injur* or verac* or trauma* or fracture* or compress* or contus* or lacerat* or transect* or lesion*)).ti. |
| 190 | exp tuberculosis/ |
| 191 | (tuberculosis or TB).ti. |
| 192 | exp urinary incontinence/ |
| 193 | urinary bladder, overactive/ |
| 194 | (bladder* adj3 (overactive* or incontin* or continence)).ti. |
| 195 | ((stress* or mixed or urg* or urin* or overflow*) adj3 (incontinen* or continence)).ti. |
| 196 | exp urticaria/ |
| 197 | urticaria*.ti. |
| 198 | chronic disease/ |
| 199 | ((chronic* or long-term) adj2 (diseas* or ill* or disorder* or condition*)).ti. |
| 200 | or/1-199 |
| 201 | exp Drug Prescriptions/ or exp Prescriptions/ |
| 202 | (Prescri* or Prescribing Behavio?r or Prescrib* Decision* or Prescribing Practice*).ti,ab. |
| 203 | (Initiat* or Start* or Commenc* or Induc*).ti. |
| 204 | or/201-203 |
| 205 | exp Clinical decision-making/ |
| 206 | exp Health Knowledge, Attitudes, Practice/ or exp Attitude of Health Personnel/ or exp Practice Patterns, Pharmacists’/ or exp Practice Patterns, Nurses’/ or exp Practice Patterns, Physicians’/ |
| 207 | (Factor* or Determinant* or Behavio?r* or Practice* or Preference* or Pattern* or Decision* or Decid* or Influenc* or Impact* or Judg* or Barrier* or Facilit* or Help or Hinder or Compl* or Accept or Conform* or Approv* or Adher* or Strateg*).ti. |
| 208 | or/205-207 |
| 209 | (Medline or systematic review).tw. or meta-analysis.pt. |
| 210 | 200 and 204 and 208 and 209 |
| 211 | limit 210 to yr=”2013-current" |

## Table 4. Web of Science search strategy

| **#** | **Web of Science Search Lines (searched 07/11/2023)** |
| --- | --- |
| 1 | TI=((vasomotor or allerg* or non-allerg* or nonallerg* or "non allerg*") and rhinitis) |
| 2 | TI=(amnesi* or "memory loss") |
| 3 | TI=(amputation or amputee*) |
| 4 | TI=an?emia |
| 5 | TI=(angina or "myocardial isch?mi*") |
| 6 | TI=(angioedema or (hereditary NEXT (angioedema* or angioNEXTotic edema*))) |
| 7 | TI=((rheumatoid or rheumatic) NEXT (arthrit* or artrit* or diseas* or condition* or nodule*)) |
| 8 | TI=("primary progressive aphasia" or aphasia or dysphasia) |
| 9 | TI=((cerebell* or spinocerebell*) NEXT (ataxia* or incoordination)) |
| 10 | TI=(addison* disease or autoimmune thyroid disease or ank* spondy* or h?emolytic an?emia or multiple sclerosis or myasthenia gravis or immune thrombocytopen* purpura or scleroderma or polyarteriti* nodosa or pernicious an?emia or vitiligo or psoriasis or primary biliary cirrhosis) |
| 11 | TI=(leukaemi* or myeloma or lymphoma) |
| 12 | TI=(stroke or posTItroke or post-stroke or cerebrovascu* or "brain vasc*" or "cerebral vasc*" or cva* or apoplexy* or sah) |
| 13 | TI=((brain* or cerebr* or vascular or cerebel* or intracran* or intracerebral* or subarachnoid) NEXT (accident or isch?mi or infarct* or thrombo* or emboli* or occlus* or h?morrhage or h?matoma* or bleed*)) |
| 14 | TI=(burn* or scald*) |
| 15 | TI=(cancer* or oncolog* or neoplasm* or carcinom* or tumo?r* or malignan* or leuk?emia) |
| 16 | TI=cardiac arrhythm* |
| 17 | TI=cerebral pals* |
| 18 | TI=(c?eliac NEXT (disease or sprue or syndrome)) |
| 19 | TI=(gluten NEXT (enteropath* or sensitiv* or hypersensitiv* or intoleran*)) |
| 20 | TI=(connective tissue disease* or sharp syndrome or polymyositis or systemic sclerosis or scleroderma) |
| 21 | TI=(heart disease* or myocardial ischemia or coronary disease* or coronary artery disease* or coronary heart disease* or myocardial infarct*) |
| 22 | TI=(allerg* or hypersensitivit* or tierg* or intolerance or anaphyla*) |
| 23 | TI=((bronchial* or respiratory or airway* or lung*) NEXT (hypersensitive* or hyperreactiv* or allerg* or insufficiency)) |
| 24 | TI=(bronch* NEXT (constrict* or spas*)) |
| 25 | TI=(asthma* or wheez* or bronchoconstrict* or antiasthma* or anti-asthma* or bronchal hyperreactivity or bronchospas*) |
| 26 | TI=((atrial or atrium) NEXT fibril?at*) |
| 27 | TI=(dravet syndrome* or "severe myoclonic epilepsy of infancy" or "severe myoclonic epilepsy in infancy" or "severe polymorphic epilepsy of infancy") |
| 28 | TI=(chronic NEXT (kidney or kidney) NEXT (disease* or failure or insufficien*)) |
| 29 | TI=(CKD or CRF or CRD or ESKD or ESRD or ESRF) |
| 30 | TI=(obstruct* NEXT (pulmonary or lung* or airway* or airflow* or bronch* or respirat*)) |
| 31 | TI=(chronic* NEXT bronchiti*) |
| 32 | TI=("chronic obstructive pulmonary disease" or "chronic obstructive airways disease" or "lung disease" or emphysema* or COPD or CAL or COAD or COLD or CBD or AECB) |
| 33 | TI=((heart or cardiac or myocardial) NEXT (failure or decompensation)) |
| 34 | TI=((congestive or acute or decompensat* or chronic) NEXT heart failure) |
| 35 | TI=((cerebr* or brain or cogniti*) NEXT (deteriorat* or insufficient* or disease or syndrome or impair*)) |
| 36 | TI=("mild cognitive impairment" or dement* or alzheimer* or binswanger* or "benign senescent forgetfulness") |
| 37 | TI=((dilated or congestive) NEXT cardiomyopath*) |
| 38 | TI=lewy* NEXT bod* |
| 39 | TI=((memory* or mental*) NEXT (declin* or deteriorat* or impair* or los*)) |
| 40 | TI=(diabet* NEXT type NEXT (one or "1" or I)) |
| 41 | TI=(diabet* NEXT type NEXT (two or "2" or II)) |
| 42 | TI=((insulin or noninsulin or non-insulin) NEXT (resistan* or depend*)) |
| 43 | TI=(diabet* or DM or DM1 or DM2 or T1D or T1DM or T2D or T2DM or NIDDM or IDDM or MODY) |
| 44 | TI=glucose ?tolerance |
| 45 | TI=(endometrios* or endometrioma? or adenomyo* or adenomyoma? or adenometriti* or adenomyosit* or adenomyometriti*) |
| 46 | TI=(epilep* or seizure* or aura* or convulsion*) |
| 47 | TI=(hypertens* or (blood NEXT pressure)) |
| 48 | TI=(chronic NEXT hepatitis) |
| 49 | TI=("hiv infect*" or "hiv disease*") |
| 50 | TI=(crohn* disease or crohn's enteritis* or ulcerative coliti* or terminal ileitis or granulomatous coliti* or ileocoliti*) |
| 51 | TI=((irritable or functional or spastic) NEXT (bowel or colon)) |
| 52 | TI=(IBS or "mucous colit*" or "gastrointestinal syndrome*" or "functional gastrointestinal") |
| 53 | TI=(migraine or megrim or hemicrania) |
| 54 | TI=("multiple sclerosis" or MS or "demyelinating disease*") |
| 55 | TI=(osteoarthr* or OA) |
| 56 | TI=(degenerative NEXT (arthritis or joint*)) |
| 57 | TI=("parkinson* disease" or PD or parkinson*) |
| 58 | TI=(mucoviscidosis or cystic fibrosis or CF or (fibrocystic disease NEXT pancrea*)) |
| 59 | TI=((gastroesophageal or gastrooesophageal or gastro ?esophageal) NEXT reflux) |
| 60 | TI=(GORD or GERD or GOR or GER) |
| 61 | TI=(gastric NEXT (acid or reflux)) |
| 62 | TI=(reflux NEXT (oesophagitis or esophagitis)) |
| 63 | TI=((peptic or gastric or duodenal or stomach or oesophageal or esophageal) and ulcer*) |
| 64 | TI=(vestibular NEXTitis or benign paroxysmal positional or BPPV or acoustic NEXToma or meniere* disease or labyrinthinitis or vestibular NEXTonitis or vertigo*) |
| 65 | TI=(eczema or atopic eczema or atopic dermatitis) |
| 66 | TI=(thyrotoxicosis* or hyperthyroid* or hypothyroid* or hypogonad* or cushing* syndrome or cushing disease or addison syndrome or addison disease or acromegaly or congenital adkidney hyperplasi*) |
| 67 | TI=(fibromyositis syndrom* or pain syndrome or fibromyalgia syndrom* or fibromyalgia* or fibrositi* or fibromyositis or rheumatism or diffuse myofascial) |
| 68 | TI=(gout or gouty arthriti* or toph* or podagra or pseudogout) |
| 69 | TI=(h?emophili* or von willebrand disease or clotting factor deficiency or clotting factor disease or hypercoagulable or thrombophilia) |
| 70 | TI=(((learning or intellectual) and (disabilit* or disorder or impairment or difficulties)) or special needs) |
| 71 | TI=((cryptogenic or idiopathic) NEXT fibrosing alveoliti*) |
| 72 | TI=("fibrocystic pulmonary dysplasia" or "idiopathic pulmon* fibros*") |
| 73 | TI=(lupus or SLE) |
| 74 | TI=(malaria or plasmodium) |
| 75 | TI=medically unexplained symptoms |
| 76 | TI=("motor neuron* disease*" or MND) |
| 77 | TI=((duchenne or pseudohypertrophic or becker or facioscapulohumeral or limb-girdle or myotonic) NEXT muscular dystrophy) |
| 78 | TI=(obes* or overweight or (over NEXT weight) or over-weight) |
| 79 | TI=((sleep* or nocturnal) NEXT (apnea* or apnoea*)) |
| 80 | TI=(sleep* NEXT disordered NEXT breathing) |
| 81 | TI=((sleep* or nocturnal) NEXT (hypopnea* or hypopnoea* or hypo-apnea* or hypo-apnoea* or apneic-hypopneic or apnoeic-hypopnoeic)) |
| 82 | TI=(OSA or SAHS or OSAHS) |
| 83 | TI=(asbestosis* or pneumoconiosis or black lung or hard metal lung disease or silicosis or talcosis) |
| 84 | TI=(osteoporo* or bone density or calcaneus) |
| 85 | TI=(amyotrophic lateral sclerosis or multiple sclerosis or huntington* disease or multiple system atrophy or prion disease) |
| 86 | TI=(peripheral arterial occlusi* or peripheral artery occlusi* or peripheral arterial isch?emi* or peripheral artery isch?emi*) |
| 87 | TI=(peripheral vascular disease* or peripheral arterial disease* or peripheral artery disease*) |
| 88 | TI=((limb* or lower extremity) NEXT isch?emi*) |
| 89 | TI=(intermittent and (occlusion or claudication)) |
| 90 | TI=(physical disabilit* or spinal cord injur* or motor learning disability* or dwarfism) |
| 91 | TI=(polycystic ovary syndrome or polycystic ovarian syndrome or PCOS) |
| 92 | TI=(progressive supranuclear pals* or PSP or richardson* syndrome or (steele richardson olsezewski NEXT (disease or syndrome)) or (steele-richardson-olsezewski NEXT (disease or syndrome))) |
| 93 | TI=psoriasis |
| 94 | TI=(rare dis* or rare diagnos* or orphan dis*) |
| 95 | TI=(sarcoid or sarcoidosis) |
| 96 | TI=(hearing loss or hearing aid* or deaf* or hearing impairment or hearing disorder) |
| 97 | TI=(visual impairment or visually impaired or visual disorder or vision impairment) |
| 98 | TI=(((h?emoglobin s disease or hbs disease or sickling disorder) and h?emoglobin s) or ((sickle cell NEXT (disease* or an?emia or disorder*)) or SCD)) |
| 99 | TI=(dyssomnia or sleep disorder*) |
| 100 | TI=((communication or learning or consciousness or perceptual or speech or voice or psychomotor) NEXT disorder*) |
| 101 | TI=(spina bifida or myelomeningocele) |
| 102 | TI=((spine or spinal or vertebr* or neck or cervical or lumbar or sacral or thoracic or cord or whiplash) NEXT2 (injur* or damag* or trauma* or fracture* or compress* or contus* or lacerat* or transect* or lesion*)) |
| 103 | TI=(tuberculosis or TB) |
| 104 | TI=(bladder* NEXT3 (overactiv* or incontin* or continence)) |
| 105 | TI=((stress* or mixed or urg* or urin* or overflow*) NEXT3 (incontinen* or continence)) |
| 106 | TI=urticaria* |
| 107 | TI=((chronic* or long-term) NEXT (diseas* or ill* or disorder* or condition*)) |
| 108 | #107 OR #106 OR #105 OR #104 OR #103 OR #102 OR #101 OR #100 OR #99 OR #98 OR #97 OR #96 OR #95 OR #94 OR #93 OR #92 OR #91 OR #90 OR #89 OR #88 OR #87 OR #86 OR #85 OR #84 OR #83 OR #82 OR #81 OR #80 OR #79 OR #78 OR #77 OR #76 OR #75 OR #74 OR #73 OR #72 OR #71 OR #70 OR #69 OR #68 OR #67 OR #66 OR #65 OR #64 OR #63 OR #62 OR #61 OR #60 OR #59 OR #58 OR #57 OR #56 OR #55 OR #54 OR #53 OR #52 OR #51 OR #50 OR #49 OR #48 OR #47 OR #46 OR #45 OR #44 OR #43 OR #42 OR #41 OR #40 OR #39 OR #38 OR #37 OR #36 OR #35 OR #34 OR #33 OR #32 OR #31 OR #30 OR #29 OR #28 OR #27 OR #26 OR #25 OR #24 OR #23 OR #22 OR #21 OR #20 OR #19 OR #18 OR #17 OR #16 OR #15 OR #14 OR #13 OR #12 OR #11 OR #10 OR #9 OR #8 OR #7 OR #6 OR #5 OR #4 OR #3 OR #2 OR #1 |
| 109 | TS=(Prescri* or Prescribing Behavio?r or Prescrib* Decision* or Prescribing Practice*) |
| 110 | TI=(Initiat* or Start* or Commenc* or Induc*) |
| 111 | #109 OR #110 |
| 112 | TI=(Factor* or Determinant* or Behavio?r* or Practice* or Preference* or Pattern* or Decision* or Decid* or Influenc* or Impact* or Judg* or Barrier* or Facilit* or Help or Hinder or Compl* or Accept or Conform* or Approv* or Adher* or Strateg*) |
| 113 | TS=(Medline or systematic review or meta-analysis) |
| 114 | #108 AND #111 AND #112 AND #113 |

## Table 5. Cochrane Library search strategy

| **#** | **Cochrane Library Search Lines (search date: 07/11/2023)** |
| --- | --- |
| 1 | MeSH descriptor: [Rhinitis] explode all trees |
| 2 | ((vasomotor or allerg* or non-allerg* or nonallerg* or non allerg*) and rhinitis):ti |
| 3 | MeSH descriptor: [Amnesia] explode all trees |
| 4 | (amnesi* or "memory loss"):ti |
| 5 | MeSH descriptor: [Amputation, Surgical] explode all trees |
| 6 | (amputation or amputee*):ti |
| 7 | MeSH descriptor: [Anemia] explode all trees |
| 8 | an?emia:ti |
| 9 | MeSH descriptor: [Cardiovascular Diseases] explode all trees |
| 10 | (angina or myocardial isch?mi*):ti |
| 11 | MeSH descriptor: [Angioedema] explode all trees |
| 12 | (angioedema or (hereditary NEXT (angioedema* or angioneurotic edema*))):ti |
| 13 | MeSH descriptor: [Arthritis] explode all trees |
| 14 | ((rheumatic or rheumatoid) NEXT (arthrit* or artrit* or diseas* or condition* or nodule*)):ti |
| 15 | MeSH descriptor: [Aphasia] explode all trees |
| 16 | ("primary progressive aphasia" or aphasia or dysphasia):ti |
| 17 | MeSH descriptor: [Ataxia] explode all trees |
| 18 | ((cerebell* or spinocerebell*) NEXT (ataxia* or incoordination)):ti |
| 19 | MeSH descriptor: [Autoimmune Diseases] explode all trees |
| 20 | (addison* disease or autoimmune thyroid disease or ank* spondy* or h?emolytic an?emia or multiple sclerosis or myasthenia gravis or immune thrombocytopen* purpura or scleroderma or polyarteriti* nodosa or pernicious an?emia or vitiligo or psoriasis or primary biliary cirrhosis):ti |
| 21 | MeSH descriptor: [Hematologic Diseases] explode all trees |
| 22 | (leukaemi* or myeloma or lymphoma):ti |
| 23 | MeSH descriptor: [Stroke] explode all trees |
| 24 | MeSH descriptor: [Brain Ischemia] explode all trees |
| 25 | MeSH descriptor: [Ischemic Attack, Transient] explode all trees |
| 26 | (stroke or poststroke or post-stroke or cerebrovascu* or brain vasc* or cerebral vasc* or cva* or apoplexy* or sah):ti |
| 27 | ((brain* or cerebr* or vascular or cerebel* or intracran* or intracerebral* or subarachnoid) NEXT (accident or isch?mi or infarct* or thrombo* or emboli* or occlus* or h?morrhage or h?matoma* or bleed*)):ti |
| 28 | MeSH descriptor: [Burns] explode all trees |
| 29 | (burn* or scald*):ti |
| 30 | MeSH descriptor: [Neoplasms] explode all trees |
| 31 | (cancer* or oncolog* or neoplasm* or carcinom* or tumo?r* or malignan* or leuk?emia):ti |
| 32 | MeSH descriptor: [Arrhythmias, Cardiac] explode all trees |
| 33 | cardiac arrhythm*:ti |
| 34 | MeSH descriptor: [Cerebral Palsy] explode all trees |
| 35 | cerebral pals*:ti |
| 36 | MeSH descriptor: [Celiac Disease] explode all trees |
| 37 | (c?eliac NEXT (disease or sprue or syndrome)):ti |
| 38 | (gluten NEXT (enteropath* or sensitiv* or hypersensitiv* or intoleran*)):ti |
| 39 | MeSH descriptor: [Connective Tissue Diseases] explode all trees |
| 40 | (connective tissue disease* or sharp syndrome or polymyositis or systemic sclerosis or scleroderma):ti |
| 41 | MeSH descriptor: [Myocardial Ischemia] explode all trees |
| 42 | (heart disease* or myocardial ischemia or coronary disease* or coronary artery disease* or coronary heart disease* or myocardial infarct*):ti |
| 43 | MeSH descriptor: [Hypersensitivity] explode all trees |
| 44 | MeSH descriptor: [Hypersensitivity] explode all trees |
| 45 | MeSH descriptor: [Allergy and Immunology] explode all trees |
| 46 | MeSH descriptor: [Anaphylaxis] explode all trees |
| 47 | (allerg* or hypersensitivit* or tierg* or intolerance or anaphyla*):ti |
| 48 | MeSH descriptor: [Asthma] explode all trees |
| 49 | MeSH descriptor: [Bronchial Spasm] explode all trees |
| 50 | MeSH descriptor: [Bronchoconstriction] explode all trees |
| 51 | ((bronchial* or respiratory or airway* or lung*) NEXT (hypersensitive* or hyperreactiv* or allerg* or insufficiency)):ti |
| 52 | (bronch* NEXT (constrict* or spas*)):ti |
| 53 | (asthma* or wheez* or bronchoconstrict* or antiasthma* or anti-asthma* or bronchal hyperreactivity or bronchospas*):ti |
| 54 | MeSH descriptor: [Atrial Fibrillation] explode all trees |
| 55 | ((atrial or atrium) NEXT fibril?at*):ti |
| 56 | MeSH descriptor: [Fatigue Syndrome, Chronic] explode all trees |
| 57 | MeSH descriptor: [Epilepsies, Myoclonic] explode all trees |
| 58 | (dravet syndrome* or "severe myoclonic epilepsy of infancy" or "severe myoclonic epilepsy in infancy" or "severe polymorphic epilepsy of infancy"):ti |
| 59 | MeSH descriptor: [Kidney Failure, Chronic] explode all trees |
| 60 | (chronic NEXT (kidney or kidney) NEXT (disease* or failure or insufficien*)):ti |
| 61 | (CKD or CRF or CRD or ESKD or ESRD or ESRF):ti |
| 62 | MeSH descriptor: [Pulmonary Disease, Chronic Obstructive] explode all trees |
| 63 | (obstruct* NEXT (pulmonary or lung* or airway* or airflow* or bronch* or respirat*)):ti |
| 64 | (chronic* NEXT bronchiti*):ti |
| 65 | ("chronic obstructive pulmonary disease" or "chronic obstructive airways disease" or "lung disease" or emphysema* or COPD or CAL or COAD or COLD or CBD or AECB):ti |
| 66 | MeSH descriptor: [Heart Failure] explode all trees |
| 67 | ((heart or cardiac or myocardial) NEXT (failure or decompensation)):ti |
| 68 | ((congestive or acute or decompensat* or chronic) NEXT heart failure):ti |
| 69 | MeSH descriptor: [Dementia] explode all trees |
| 70 | ((cerebr* or brain or cogniti*) NEXT (deteriorat* or insufficient* or disease or syndrome or impair*)):ti |
| 71 | ("mild cognitive impairment" or dement* or alzheimer* or binswanger* or "benign senescent forgetfulness"):ti |
| 72 | ((dilated or congestive) NEXT cardiomyopath*):ti |
| 73 | (lewy* NEXT bod*):ti |
| 74 | ((memory* or mental*) NEXT (declin* or deteriorat* or impair* or los*)):ti |
| 75 | MeSH descriptor: [Diabetes Mellitus] explode all trees |
| 76 | (diabet* NEXT3 type NEXT (one or "1" or I)):ti |
| 77 | (diabet* NEXT3 type NEXT (two or "2" or II)):ti |
| 78 | ((insulin or noninsulin or non-insulin) NEXT (resistan* or depend*)):ti |
| 79 | (diabet* or DM or DM1 or DM2 or T1D or T1DM or T2D or T2DM or NIDDM or IDDM or MODY):ti |
| 80 | glucose ?tolerance:ti |
| 81 | MeSH descriptor: [Endometriosis] explode all trees |
| 82 | (endometrios* or endometrioma? or adenomyo* or adenomyoma? or adenometriti* or adenomyosit* or adenomyometriti*):ti |
| 83 | MeSH descriptor: [Epilepsy] explode all trees |
| 84 | MeSH descriptor: [Seizures] explode all trees |
| 85 | (epilep* or seizure* or aura* or convulsion*):ti |
| 86 | MeSH descriptor: [Hypertension] explode all trees |
| 87 | (hypertens* or (blood NEXT pressure)):ti |
| 88 | MeSH descriptor: [Hepatitis, Chronic] explode all trees |
| 89 | (chronic NEXT hepatitis):ti |
| 90 | MeSH descriptor: [HIV] explode all trees |
| 91 | (hiv infect* or hiv disease*):ti |
| 92 | MeSH descriptor: [Inflammatory Bowel Diseases] explode all trees |
| 93 | (crohn* disease or crohn's enteritis* or ulcerative coliti* or terminal ileitis or granulomatous coliti* or ileocoliti*):ti |
| 94 | MeSH descriptor: [Irritable Bowel Syndrome] explode all trees |
| 95 | ((irritable or functional or spastic) NEXT (bowel or colon)):ti |
| 96 | (IBS or mucous colit* or gastrointestinal syndrome* or functional gastrointestinal):ti |
| 97 | MeSH descriptor: [Migraine Disorders] explode all trees |
| 98 | (migraine or megrim or hemicrania):ti |
| 99 | MeSH descriptor: [Multiple Sclerosis] explode all trees |
| 100 | (multiple sclerosis or MS or demyelinating disease*):ti |
| 101 | MeSH descriptor: [Osteoarthritis] explode all trees |
| 102 | (osteoarthr* or OA):ti |
| 103 | (degenerative NEXT (arthritis or joint*)):ti |
| 104 | MeSH descriptor: [Parkinson Disease] explode all trees |
| 105 | (parkinson* disease or PD or parkinson*):ti |
| 106 | MeSH descriptor: [Cystic Fibrosis] explode all trees |
| 107 | (cystic fibrosis or CF or mucoviscidosis or (fibrocystic disease NEXT pancrea*)):ti |
| 108 | MeSH descriptor: [Gastroesophageal Reflux] explode all trees |
| 109 | MeSH descriptor: [Ulcer] explode all trees |
| 110 | MeSH descriptor: [Esophagitis] explode all trees |
| 111 | ((gastroesophageal or gastrooesophageal or gastro ?esophageal) NEXT reflux):ti |
| 112 | (GORD or GERD or GOR or GER):ti |
| 113 | (gastric NEXT (acid or reflux)):ti |
| 114 | (reflux NEXT (oesophagitis or esophagitis)):ti |
| 115 | ((peptic or gastric or duodenal or stomach or oesophageal or esophageal) and ulcer*):ti |
| 116 | MeSH descriptor: [Dizziness] explode all trees |
| 117 | MeSH descriptor: [Vertigo] explode all trees |
| 118 | (vestibular neuritis or benign paroxysmal positional or BPPV or acoustic neuroma or meniere* disease or labyrinthinitis or vestibular neuronitis or vertigo*):ti |
| 119 | MeSH descriptor: [Eczema] explode all trees |
| 120 | (eczema or atopic eczema or atopic dermatitis):ti |
| 121 | MeSH descriptor: [Endocrine System Diseases] explode all trees |
| 122 | (thyrotoxicosis* or hyperthyroid* or hypothyroid* or hypogonad* or cushing* syndrome or cushing disease or addison syndrome or addison disease or acromegaly or congenital adkidney hyperplasi*):ti |
| 123 | MeSH descriptor: [Fibromyalgia] explode all trees |
| 124 | (fibromyositis syndrom* or pain syndrome or fibromyalgia syndrom* or fibromyalgia* or fibrositi* or fibromyositis or rheumatism or diffuse myofascial):ti |
| 125 | MeSH descriptor: [Gout] explode all trees |
| 126 | (gout or gouty arthriti* or toph* or podagra or pseudogout):ti |
| 127 | MeSH descriptor: [Blood Coagulation Disorders] explode all trees |
| 128 | (h?emophili* or von willebrand disease or clotting factor deficiency or clotting factor disease or hypercoagulable or thrombophilia):ti |
| 129 | MeSH descriptor: [Learning Disabilities] explode all trees |
| 130 | (((learning or intellectual) and (disabilit* or disorder or impairment or difficulties)) or special needs):ti |
| 131 | MeSH descriptor: [Pulmonary Fibrosis] explode all trees |
| 132 | ((cryptogenic or idiopathic) NEXT fibrosing alveoliti*):ti |
| 133 | (fibrocystic pulmonary dysplasia or idiopathic pulmon* fibros*):ti |
| 134 | MeSH descriptor: [Lupus Erythematosus, Systemic] explode all trees |
| 135 | (lupus or SLE):ti |
| 136 | MeSH descriptor: [Malaria] explode all trees |
| 137 | (malaria or plasmodium):ti |
| 138 | MeSH descriptor: [Medically Unexplained Symptoms] explode all trees |
| 139 | medically unexplained symptoms:ti |
| 140 | MeSH descriptor: [Motor Neuron Disease] explode all trees |
| 141 | (motor neuron* disease* or MND):ti |
| 142 | MeSH descriptor: [Muscular Dystrophies] explode all trees |
| 143 | ((duchenne or pseudohypertrophic or becker or facioscapulohumeral or limb-girdle or myotonic) NEXT muscular dystrophy):ti |
| 144 | MeSH descriptor: [Obesity] explode all trees |
| 145 | (obes* or overweight or (over NEXT weight) or over-weight):ti |
| 146 | MeSH descriptor: [Sleep Apnea, Obstructive] explode all trees |
| 147 | ((sleep* or nocturnal) NEXT (apnea* or apnoea*)):ti |
| 148 | (sleep* NEXT disordered NEXT breathing):ti |
| 149 | ((sleep* or nocturnal) NEXT (hypopnea* or hypopnoea* or hypo-apnea* or hypo-apnoea* or apneic-hypopneic or apnoeic-hypopnoeic)):ti |
| 150 | (OSA or SAHS or OSAHS):ti |
| 151 | MeSH descriptor: [Asthma, Occupational] explode all trees |
| 152 | (asbestosis* or pneumoconiosis or black lung or hard metal lung disease or silicosis or talcosis):ti |
| 153 | MeSH descriptor: [Osteoporosis] explode all trees |
| 154 | (osteoporo* or bone density or calcaneus):ti |
| 155 | MeSH descriptor: [Neurodegenerative Diseases] explode all trees |
| 156 | (amyotrophic lateral sclerosis or multiple sclerosis or huntington* disease or multiple system atrophy or prion disease):ti |
| 157 | MeSH descriptor: [Peripheral Vascular Diseases] explode all trees |
| 158 | (peripheral arterial occlusi* or peripheral artery occlusi* or peripheral arterial isch?emi* or peripheral artery isch?emi*):ti |
| 159 | (peripheral vascular disease* or peripheral arterial disease* or peripheral artery disease*):ti |
| 160 | ((limb* or lower extremity) NEXT isch?emi*):ti |
| 161 | (intermittent and (occlusion or claudication)):ti |
| 162 | MeSH descriptor: [Disabled Persons] explode all trees |
| 163 | (physical disabilit* or spinal cord injur* or motor learning disability* or dwarfism):ti |
| 164 | MeSH descriptor: [Polycystic Ovary Syndrome] explode all trees |
| 165 | (polycystic ovary syndrome or polycystic ovarian syndrome or PCOS):ti |
| 166 | MeSH descriptor: [Supranuclear Palsy, Progressive] explode all trees |
| 167 | (progressive supranuclear pals* or PSP or richardson* syndrome or (steele richardson olsezewski NEXT (disease or syndrome)) or (steele-richardson-olsezewski NEXT (disease or syndrome))):ti |
| 168 | MeSH descriptor: [Psoriasis] explode all trees |
| 169 | psoriasis:ti |
| 170 | MeSH descriptor: [Rare Diseases] explode all trees |
| 171 | (rare dis* or rare diagnos* or orphan dis*):ti |
| 172 | MeSH descriptor: [Sarcoidosis] explode all trees |
| 173 | (sarcoid or sarcoidosis):ti |
| 174 | MeSH descriptor: [Deafness] explode all trees |
| 175 | MeSH descriptor: [Blindness] explode all trees |
| 176 | MeSH descriptor: [Visually Impaired Persons] explode all trees |
| 177 | (hearing loss or hearing aid* or deaf* or hearing impairment or hearing disorder):ti |
| 178 | (visual impairment or visually impaired or visual disorder or vision impairment):ti |
| 179 | MeSH descriptor: [Anemia, Sickle Cell] explode all trees |
| 180 | (((h?emoglobin s disease or hbs disease or sickling disorder) and h?emoglobin s) or ((sickle cell NEXT (disease* or an?emia or disorder*)) or SCD)):ti |
| 181 | MeSH descriptor: [Skin Diseases] explode all trees |
| 182 | MeSH descriptor: [Dyssomnias] explode all trees |
| 183 | (dyssomnia or sleep disorder*):ti |
| 184 | MeSH descriptor: [Speech Disorders] explode all trees |
| 185 | ((communication or learning or consciousness or perceptual or speech or voice or psychomotor) NEXT disorder*):ti |
| 186 | MeSH descriptor: [Spina Bifida Occulta] explode all trees |
| 187 | (spina bifida or myelomeningocele):ti |
| 188 | MeSH descriptor: [Spinal Injuries] explode all trees |
| 189 | ((spine or spinal or vertebr* or neck or cervical or lumbar or sacral or thoracic or cord or whiplash) NEXT (injur* or damag* or trauma* or fracture* or compress* or contus* or lacerat* or transect* or lesion*)):ti |
| 190 | MeSH descriptor: [Tuberculosis] explode all trees |
| 191 | (tuberculosis or TB):ti |
| 192 | MeSH descriptor: [Urinary Incontinence] explode all trees |
| 193 | MeSH descriptor: [Urinary Bladder, Overactive] explode all trees |
| 194 | (bladder* NEXT (overactiv* or incontin* or continence)):ti |
| 195 | ((stress* or mixed or urg* or urin* or overflow*) NEXT (incontinen* or continence)):ti |
| 196 | MeSH descriptor: [Urticaria] explode all trees |
| 197 | urticaria*:ti |
| 198 | MeSH descriptor: [Chronic Disease] explode all trees |
| 199 | ((chronic* or long-term) NEXT (diseas* or ill* or disorder* or condition*)):ti |
| 200 | 1 or 2 or 3 or 4 or 5 or 6 or 7 or 8 or 9 or 10 or 11 or 12 or 13 or 14 or 15 or 16 or 17 or 18 or 19 or 20 or 21 or 22 or 23 or 24 or 25 or 26 or 27 or 28 or 29 or 30 or 31 or 32 or 33 or 34 or 35 or 36 or 37 or 38 or 39 or 40 or 41 or 42 or 43 or 44 or 45 or 46 or 47 or 48 or 49 or 50 or 51 or 52 or 53 or 54 or 55 or 56 or 57 or 58 or 59 or 60 or 61 or 62 or 63 or 64 or 65 or 66 or 67 or 68 or 69 or 70 or 71 or 72 or 73 or 74 or 75 or 76 or 77 or 78 or 79 or 80 or 81 or 82 or 83 or 84 or 85 or 86 or 87 or 88 or 89 or 90 or 91 or 92 or 93 or 94 or 95 or 96 or 97 or 98 or 99 or 100 or 101 or 102 or 103 or 104 or 105 or 106 or 107 or 108 or 109 or 110 or 111 or 112 or 113 or 114 or 115 or 116 or 117 or 118 or 119 or 120 or 121 or 122 or 123 or 124 or 125 or 126 or 127 or 128 or 129 or 130 or 131 or 132 or 133 or 134 or 135 or 136 or 137 or 138 or 139 or 140 or 141 or 142 or 143 or 144 or 145 or 146 or 147 or 148 or 149 or 150 or 151 or 152 or 153 or 154 or 155 or 156 or 157 or 158 or 159 or 160 or 161 or 162 or 163 or 164 or 165 or 166 or 167 or 168 or 169 or 170 or 171 or 172 or 173 or 174 or 175 or 176 or 177 or 178 or 179 or 180 or 181 or 182 or 183 or 184 or 185 or 186 or 187 or 188 or 189 or 190 or 191 or 192 or 193 or 194 or 195 or 196 or 197 or 198 or 199 |
| 201 | MeSH descriptor: [Drug Prescriptions] explode all trees |
| 202 | MeSH descriptor: [Prescriptions] explode all trees |
| 203 | (Prescri* or Prescribing Behavio?r or Prescrib* Decision* or Prescribing Practice*):ti,ab |
| 204 | (Initiat* or Start* or Commenc* or Induc*):ti |
| 205 | 201 or 202 or 203 or 204 |
| 206 | MeSH descriptor: [Clinical Decision-Making] explode all trees |
| 207 | MeSH descriptor: [Health Knowledge, Attitudes, Practice] explode all trees |
| 208 | MeSH descriptor: [Attitude of Health Personnel] explode all trees |
| 209 | MeSH descriptor: [Practice Patterns, Physicians'] explode all trees |
| 210 | MeSH descriptor: [Practice Patterns, Pharmacists'] explode all trees |
| 211 | MeSH descriptor: [Practice Patterns, Nurses'] explode all trees |
| 212 | (Factor* or Determinant* or Behavio?r* or Practice* or Preference* or Pattern* or Decision* or Decid* or Influenc* or Impact* or Judg* or Barrier* or Facilit* or Help or Hinder or Compl* or Accept or Conform* or Approv* or Adher* or Strateg*):ti |
| 213 | 206 or 207 or 208 or 209 or 210 or 211 or 212 |
| 214 | (Medline or systematic review or meta-analysis):ti,ab |
| 215 | 200 and 205 and 213 and 214 |

## Table 6. Google Scholar search strategy

| **Google Scholar (search date: 10/10/2023)** |
| --- |
| Search for review articles between 2013 and 2023, limited to the first 200 studies |
| “prescribing OR prescriptions AND factor OR factors OR determinant OR determinants OR barrier OR barriers OR facilitator OR facilitators OR influence OR influencing OR decision OR deciding OR behaviour OR attitudes AND systematic review OR meta-review OR meta-analysis OR meta review OR meta analysis OR review -antibiotic -non-medical -opioid” |
