# Supplementary figures and images for: A conceptual model of factors potentially influencing prescribing decisions for chronic conditions: an overview of systematic reviews
Source: BMC Med. 2025 Jul 1;23:364. doi: 10.1186/s12916-025-04194-9 (PMC12217990; doi:10.1186/s12916-025-04194-9)

##
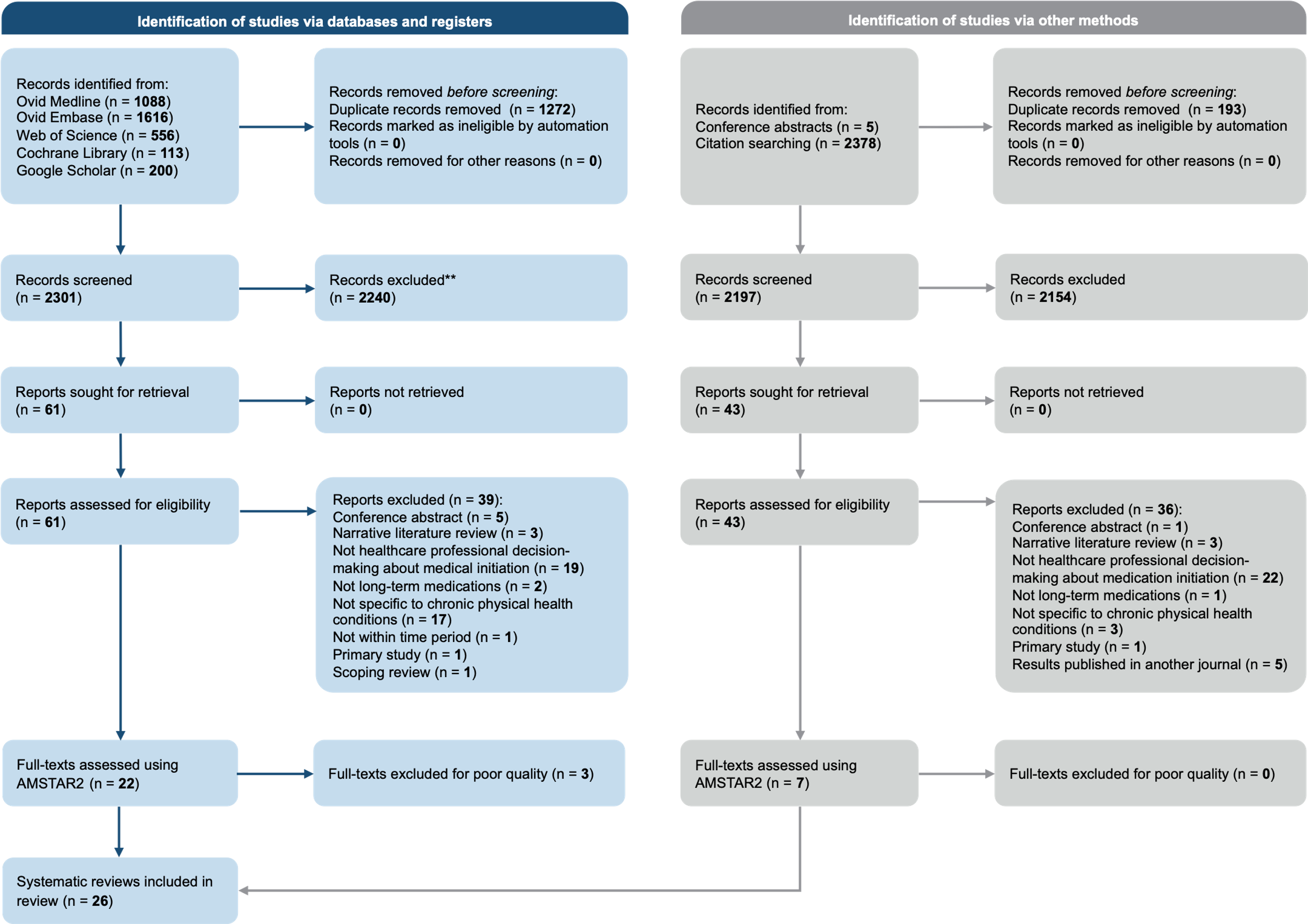
Figure 1. Detailed PRISMA diagram

Supplement: Supplementary file 3 — Additional file 3: Fig. 1 Detailed PRISMA diagram. [file 12916_2025_4194_MOESM3_ESM.docx]
