# Supplementary material for: A conceptual model of factors potentially influencing prescribing decisions for chronic conditions: an overview of systematic reviews
Source: BMC Med. 2025 Jul 1;23:364. doi: 10.1186/s12916-025-04194-9 (PMC12217990; doi:10.1186/s12916-025-04194-9)
Supplement: Supplementary file 4 — Additional file 4: Table 7 Detailed summary of included studies. [file 12916_2025_4194_MOESM4_ESM.docx]

## Table 7. Detailed summary of included studies

| **Study Year** | **Country of origin** | **Databases (n)** | **Date range of search** | **Publication date range** | **Studies (n)** | **Origin of studies** | **Setting** | **Disease area** | **Study type** | **Funding source** |
| --- | --- | --- | --- | --- | --- | --- | --- | --- | --- | --- |
| Abdelkader 2023 | Qatar | 10 | Jan 2010 – Mar 2021 | 2010 – 2020 | 40 | International | Primary, Secondary | Hypertension | Mixed | University |
| Arshad 2020 | Pakistan | 3 | Jan 2000 – Jan 2019 | 2000 – 2018 | 26 | International | Primary, Secondary | Hypertension | Quantitative | None |
| Bin Rsheed 2017 | Saudi Arabia | 8 | Jan 1994 – August 2014 | 2005 – 2014 | 19 | International | Primary, Secondary | Diabetes | Mixed | Not reported |
| Byrne 2022 | United Kingdom | 8 | Jan 2006 – Feb 2021 | Not reported | 42 | International | Primary, Secondary | Diabetes | Qualitative | Pharmaceutical company |
| Chin 2016 | Australia | 4 | Jan 2000 – Jan 2015 | 1994 – 2012 | 23 | International | Secondary | Cardiovascular disease | Quantitative | None |
| Dhungana 2021 | Nepal | 6 | Jan 2000 – Jan 2020 | Not reported | 15 | Nepal | Primary, Secondary | Hypertension | Mixed | None |
| Generalova 2018 | United Kingdom | 4 | Unknown | 2013 – 2016 | 10 | International | Primary, Secondary | Atrial fibrillation | Mixed | None |
| Iudici 2014 | Italy | 1 | Jan 2000 – Mar 2013 | 2005 – 1013 | 23 | International | Secondary | Systemic sclerosis | Quantitative | None |
| Ju 2018 | Australia | 5 | Inception – Apr 2018 | 1997 – 2017 | 34 | International | Primary | Cardiovascular disease | Mixed | Not reported |
| Kaushik 2020 | India | 3 | Unknown | Not reported | 13 | USA | Primary | Diabetes | Mixed | Pharmaceutical company |
| Khatib 2022 | Canada | 3 | Inception – Feb 2013 | Not reported | 69 | International | Primary, Secondary | Hypertension | Mixed | None |
| Lalor 2022 | Australia | 3 | Inception – Jun 2021 | 2004 – 2019 | 15 | International | Secondary | Inflammatory arthritis | Mixed | Governmental body |
| Mahmoud 2023 | United Kingdom | 4 | Jan 2009 – Jan 2021 | 2013 – 2023 | 40 | International | Primary, Secondary | Diabetes | Quantitative | University |
| Maimaris 2013 | United Kingdom | 7 | Inception – May 2013 | 1992 – 2011 | 53 | International | Primary, Secondary | Hypertension | Mixed | Research organisation |
| Mas Dalmau 2017 | Spain | **4** | Inception – May 2013 | 1994 – 2012 | 9 | International | Primary, Secondary | Atrial fibrillation | Mixed | Governmental body |
| Ng 2015 | Malaysia | 4 | Inception – Jun 2014 | Not reported | 25 | International | Primary, Secondary | Diabetes | Mixed | Research organisation |
| Oqab 2018 | Canada | 3 | Unknown | 2009 – 2015 | 3 | International | Primary, Secondary | Atrial fibrillation | Quantitative | None |
| Orayj 2019 | United Kingdom | 4 | Jan 1967 – Mar 2018 | Not reported | 44 | International | Primary, Secondary | Parkinson’s disease | Mixed | University |
| Osasu 2021 | United Kingdom | 4 | Jan 1990 – Jan 2018 | 1995 – 2018 | 34 | International | Primary, Secondary | Atrial fibrillation | Mixed | None |
| Pokorny 2021 | Australia | 4 | Inception – Feb 2021 | 2007 – 2021 | 31 | International | Secondary | Cancer | Quantitative | University |
| Presta 2022 | Italy | 2 | Inception – May 2022 | 2008 – 2022 | 23 | International | Primary, Secondary | Atrial fibrillation | Quantitative | None |
| Pritchett 2020 | United Kingdom | 4 | Inception – Jun 2018 | 2001 – 2019 | 13 | International | Primary, Secondary, Tertiary | Atrial fibrillation | Mixed | Governmental body |
| Proietti 2022 | Italy | 2 | Inception – Sep 2021 | 2009 – 2021 | 33 | International | Primary, Secondary | Atrial fibrillation | Quantitative | None |
| Qadi 2020 | United Kingdom | 9 | Inception – Sep 2018 | 2011 – 2015 | 5 | Europe | Primary | Cardiovascular disease | Qualitative | University |
| Rushforth 2014 | United Kingdom | 5 | Jan 1980 – Mar 2014 | 1998 – 2014 | 32 | International | Primary | Diabetes | Qualitative | None |
| Wilkinson 2019 | United Kingdom | 5 | Inception – Oct 2017 | 2008 – 2017 | 20 | International | Primary, Secondary | Atrial fibrillation | Quantitative | Research organisation |
