# Supplementary material for: A conceptual model of factors potentially influencing prescribing decisions for chronic conditions: an overview of systematic reviews
Source: BMC Med. 2025 Jul 1;23:364. doi: 10.1186/s12916-025-04194-9 (PMC12217990; doi:10.1186/s12916-025-04194-9)
Supplement: Supplementary file 5 — Additional file 5: Table 8 Excluded studies and reasons at full-text screening. [file 12916_2025_4194_MOESM5_ESM.docx]

## Table 8. Excluded studies and reasons at full-text screening

| **Author (year)** | **Title (limited to 4 words)** | **Reason for exclusion** |
| --- | --- | --- |
| Al et al (2022) | Prevalence, contributory factors and | Not healthcare professional decision-making about medication initiation |
| Albitar et al (2020) | Adherence to Global Initiative | Narrative literature review |
| Alhawassi et al (2015) | Hypertension in older persons | Not healthcare professional decision-making about medication initiation |
| Bain et al (2019) | Interventions to improve insulin | Not healthcare professional decision-making about medication initiation |
| Bajorek et al (2007) | Management of warfarin in | Primary study |
| Bakr et al (2018) | A systematic review of | Not healthcare professional decision-making about medication initiation |
| Blasco-Blasco et al (2020) | Barrier and facilitators to | Not healthcare professional decision-making about medication initiation |
| Bo et al (2017) | Oral anticoagulant therapy for | Narrative literature review |
| Bora et al (2023) | The effectiveness of clinical | Not healthcare professional decision-making about medication initiation |
| Borzecki et al (2005) | Barriers to hypertension control | Not healthcare professional decision-making about medication initiation |
| Brax et al (2017) | Association between physicians' interaction | Not specific to chronic physical health conditions |
| Brundisini et al (2015) | Type 2 diabetes patients' | Not healthcare professional decision-making about medication initiation |
| Bul et al (2022) | Frailty and oral anticoagulant | Not healthcare professional decision-making about medication initiation |
| Bul et al (2022) | Frailty and oral anticoagulant | Results published in another journal |
| Bungard et al (2000) | Why do patients with | Narrative literature review |
| Dhungana et al (2021) | Trends in the prevalence | Not healthcare professional decision-making about medication initiation |
| Egerton et al (2017) | A systematic review and | Not long-term medications |
| Ettehad et al (2016) | Blood pressure lowering for | Not healthcare professional decision-making about medication initiation |
| Fahey et al (2005) | Educational and organisational interventions | Not healthcare professional decision-making about medication initiation |
| Fickweiler et al (2017) | Interactions between physicians and | Not specific to chronic physical health conditions |
| Gabbay et al (2020) | Addressing Therapeutic Inertia in | Narrative literature review |
| Gebreyohannes et al (2021) | Thromboprophylaxis for patients with | Conference abstract only |
| Gebreyohannes et al (2021) | Non-adherence to thromboprophylaxis guidelines | Narrative literature review |
| Glynn et al (2010) | Self-monitoring and other non-pharmacological | Not healthcare professional decision-making about medication initiation |
| Griffin et al (2017) | Impact of metformin on | Not healthcare professional decision-making about medication initiation |
| Hagen et al (2016) | Quality of community-based osteoarthritis | Not healthcare professional decision-making about medication initiation |
| Hart et al (2007) | Meta-analysis: antithrombotic therapy to | Not healthcare professional decision-making about medication initiation |
| He et al (2022) | Impact of frailty on | Not healthcare professional decision-making about medication initiation |
| Huang et al (2013) | Impact of pay-for-performance on | Not healthcare professional decision-making about medication initiation |
| Iudici et al (2013) | Glucocorticoids in systemic sclerosis | Results published in another journal |
| Jarrar et al (2021)[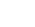](http://libkey.io/https:/dx.doi.org/10.1007/s11096-021-01269-4)[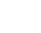](https://scholar.google.com/scholar?q=Thromboprophylaxis+for+patients+with+atrial+fibrillation%3A+A+systematic+review+of+strategies+to+improve+guideline+adherence+in+primary+care) | Factors influencing oral anticoagulant | Conference abstract only |
| Jeyaruban et al (2015) | Management of gout in | Not healthcare professional decision-making about medication initiation |
| Kataoka et al (2020) | Cumulative network-meta-analyses, practice | Not healthcare professional decision-making about medication initiation |
| Kaushik et al (2020) | Challenges in insulin initiation | Not healthcare professional decision-making about medication initiation |
| Lam et al (2022) | Prescribing behavior of antidepressants | Not specific to chronic physical health conditions |
| Lotfi et al (2016) | Knowledge, beliefs and attitudes | Not specific to chronic physical health conditions |
| Lundby et al (2019) | Health care professionals' attitudes | Not healthcare professional decision-making about medication initiation |
| Malik et al (2019) | Meta-analysis of direct-acting | Not healthcare professional decision-making about medication initiation |
