# Supplementary material for: A conceptual model of factors potentially influencing prescribing decisions for chronic conditions: an overview of systematic reviews
Source: BMC Med. 2025 Jul 1;23:364. doi: 10.1186/s12916-025-04194-9 (PMC12217990; doi:10.1186/s12916-025-04194-9)
Supplement: Supplementary file 6 — Additional file 6: Table 9 Detailed AMSTAR 2 quality assessment. [file 12916_2025_4194_MOESM6_ESM.docx]

## Table 9. Detailed AMSTAR2 quality assessment

Index: Green - Yes, Yellow - Partially yes, Red - No, Blue - Unclear, Grey - Not applicable; L - Low, CL - Critically low
